# Dynamics of the Mammalian Placental Metabolome in Placentogenesis and Embryonic Development

**DOI:** 10.1002/advs.202507928

**Published:** 2026-01-28

**Authors:** Gang Chen, Zichen Liu, Mengyi Wei, Qian Li, Liang Wu, Kunyuan Yu, Yanhong Xu, Dainan Yu, Wenwu Ma, Hongmei Wang, Ng Shyh‐Chang, Jinglei Zhai

**Affiliations:** ^1^ State Key Laboratory of Organ Regeneration and Reconstruction Institute of Zoology Chinese Academy of Sciences Beijing P. R. China; ^2^ Institute of Molecular Medicine College of Future Technology Peking University Beijing China; ^3^ University of Chinese Academy of Sciences Beijing P. R. China; ^4^ Beijing Institute for Stem Cell and Regenerative Medicine Beijing P. R. China; ^5^ Reproductive Medical Center the First Affiliated Hospital of Zhengzhou University Zhengzhou Henan P. R. China; ^6^ Henan Key Laboratory of Reproduction and Genetics the First Affiliated Hospital of Zhengzhou University Zhengzhou Henan P. R. China

**Keywords:** embryo in vitro culture, embryonic development, nicotinamide adenine dinucleotide, placenta metabolism, psm‐like progenitor cells

## Abstract

Mammalian placental metabolism is crucial for both placental and embryonic development. However, the metabolic profiles of placentas and their regulatory roles in placentogenesis and embryonic development at different developmental stages remain poorly characterized. To address these questions, we collect 501 mouse placentas spanning embryonic day (E) 8.5–14.5 and construct metabolomic–transcriptomic atlases of placentogenesis. Metabolomic and transcriptomic analyses reveal that placental samples from E8.5 to E14.5 are clustered into three separated states: E8.5, E9.5–10.5, and E11.5–14.5, pinpointing the metabolic transitions during placentogenesis from E8.5 to E9.5 and from E10.5 to E11.5. Based on a series of metabolite and enrichment analyses, Nicotinamide adenine dinucleotide (NAD(H)), flavin adenine dinucleotide (FAD), and L‐glutamate (Glu) are identified as differentially abundant metabolites (DAMs) during E8.5–14.5. Using in vitro cultured (IVC) embryos, NAD(H) is shown to promote the extension of embryonic body length, through accelerated segmentation and increased proliferation, as verified in NAD(H)‐treated mouse embryonic stem cell (mESC)‐induced presomitic mesoderm (PSM)‐like progenitor cells. These findings not only serve as an invaluable resource for understanding placental metabolism and its contribution to embryogenesis but also shed light on the mechanisms underlying developmental abnormalities associated with placental metabolic dysfunction.

## Graphical abstract

This study identifies three metabolic stages (E8.5, E9.5–10.5, E11.5–14.5) and two transition periods (E8.5–9.5, E10.5–11.5) in mouse placental development. NAD(H) emerges as a key dynamic metabolite that enhances embryonic growth through accelerated segmentation and increased proliferation of mouse embryonic stem cell (mESC)‐induced presomitic mesoderm (PSM)‐like progenitor cells. These findings suggest that NAD(H) has the potential to optimize embryoids and organoids.

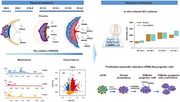

## 1 Introduction

The mammalian placenta is an essential organ with multiple functions, including nutrient and waste exchange, immune tolerance, and protection against pathogens. Mouse placentogenesis initiates as early as the 4‐cell stage during the first pro‐trophectoderm (TE) cell‐fate specification events in the embryo or conceptus [1, 2, 3]. Subsequent cell divisions lead to the formation of a blastocyst at E3.5. The blastocyst consists of the outer TE, the inner cell mass (ICM), and the blastocoel cavity. ICM then separates into the epiblast (EPI) and the primitive endoderm (PrE). The polar TE covers the EPI at the embryonic pole, while the mural TE overlays the blastocoel at the abembryonic pole [4]. At E4.0, the mural TE differentiates into primary parietal trophoblast giant cells (primary P‐TGCs) for implantation into the uterus, while the polar TE gives rise to the extra‐embryonic ectoderm (ExE). The persistent expansion of the ExE generates the ectoplacental cone (EPC, Figure S1a). Then, the embryo or conceptus extends along the proximal and distal axis during E5.0–6.0. Starting from E6.5, the embryo undergoes gastrulation, and the gastrulating cells on the posterior side of EPI contribute to the extra‐embryonic mesoderm (ExM), which then develops into the allantois around E7.5 [5]. Then, the basal region of the EPC cavity and the ExM together form the chorionic plate, which makes attachment with the allantois at E8.5, establishing the placenta anlage [6, 7]. From E8.5 to E10.5, ExE‐derived chorionic trophoblast cells begin to differentiate into two layers of multinucleated syncytiotrophoblast cells (SynT‐I and SynT‐II) through cell‐cell fusion, and a layer of mononucleated sinusoid TGCs (S‐TGCs) [8]. EPC cells overlaying the labyrinth layer further differentiate into spongiotrophoblast (SpT), glycogen cells (GCs), and multiple TGC subtypes, including channel TGCs (Ch‐TGCs), canal TGCs (C‐TGCs), spiral artery‐associated TGCs (SpA‐TGCs), and secondary P‐TGCs, which together form the junctional zone [9]. The mature mouse placenta is established around E10.5, which consists of three main layers: the labyrinth, the junctional zone, and the maternal decidua. From E10.5 to E12.5, the labyrinth and decidua regions expand, while the junctional zone contracts [10, 11]. Morphological and functional stability of the mouse placenta is established at E14.5 and persists until birth [12] (Figure S1a).

The mouse embryo or conceptus initially relies on histiotrophic nutrition during early development (approximately E5.0–8.5), when the embryo has not yet established a connection with the maternal blood circulation, and thus depends on placental trophoblast‐mediated uptake of tissue fluids and uterine gland secretions [13, 14]. By E8.5, fusion of the allantois with the chorionic plate forms the chorioallantoic placenta, providing the anatomical basis for maternal‐placental‐fetal blood contact. By E10.5, the chorioallantoic placenta has been vascularized, allowing the fetal blood to perfuse into the placental labyrinth. At the same time, maternal blood also begins to perfuse the placental labyrinth, thus establishing the blood‐mediated exchange interface and initiating the transition from histiotrophic to hemotrophic nutrition. This process indicates the key timepoints at which we expect potential changes in the metabolic and energetic features of the developing placenta.

The placental metabolome undergoes significant changes at various stages of placentogenesis and is essential for placenta function and embryonic development. For example, polyamine depletion in trophoblasts reduces both glycolysis and oxidative phosphorylation, resulting in decreased acetyl‐CoA availability and global histone hypoacetylation in a sex‐dependent manner [15]. Placenta‐secreted 5‐hydroxytryptamine (5‐HT) is a crucial modulator for fetal brain development, affecting neurotransmitter precursor supply, receptor‐mediated signaling, and neurotrophic interactions [16]. Extracellular vesicles (EVs) derived from mature placental trophoblasts, which carry lipids, proteins, and nucleic acids, enhance fetal cardiomyocyte development and maturation [17]. However, the existing research has mainly focused on certain placental formation stages, which is insufficient to offer comprehensive and consecutive information for understanding the placenta's metabolic features and their effects on embryonic development.

To elucidate the dynamic placental metabolome during placentogenesis and its effects on embryonic development, we collected mouse placenta samples at E8.5–14.5 for metabolome and transcriptome sequencing (Table S1). Based on metabolome and transcriptome analyses, placentas from E8.5 to E14.5 can be stage‐specifically clustered into three states: E8.5, E9.5–10.5, and E11.5–14.5, pinpointing the two dramatic transitions from E8.5 to E9.5 and from E10.5 to E11.5. We also identified that nicotinamide adenine dinucleotide (NAD(H)), flavin adenine dinucleotide (FAD), and L‐glutamate (Glu) are differentially abundant metabolites (DAMs), which dramatically changed in placentas from E8.5 to E14.5, especially at the first transition E8.5‐9.5. To explore whether the acute increase in placental NAD(H) affects embryonic development, we cultured mouse embryos in vitro with the addition of NAD(H). The results showed that NAD(H) promoted the extension of embryonic body length, which might result from accelerated segmentation and enhanced cell proliferation, verified by the NAD(H)‐treated mouse embryonic stem cell (mESC)‐induced presomitic mesoderm (PSM)‐like progenitor cells. Our findings offer valuable insights into the role of placenta metabolism, which may expand our understanding of the metabolic influence that underpins maternal‐fetal nutrient exchange in physiological and pathological situations, and also assist us in establishing more robust and longer‐term placental organoids, embryos, and embryoids for in vitro culture (IVC) systems.

## 2 Results

### 2.1 High‐Resolution Metabolomic Atlas Reveals Dynamic Metabolic Changes in Mouse E8.5–14.5 Placentas

**FIGURE 1:**
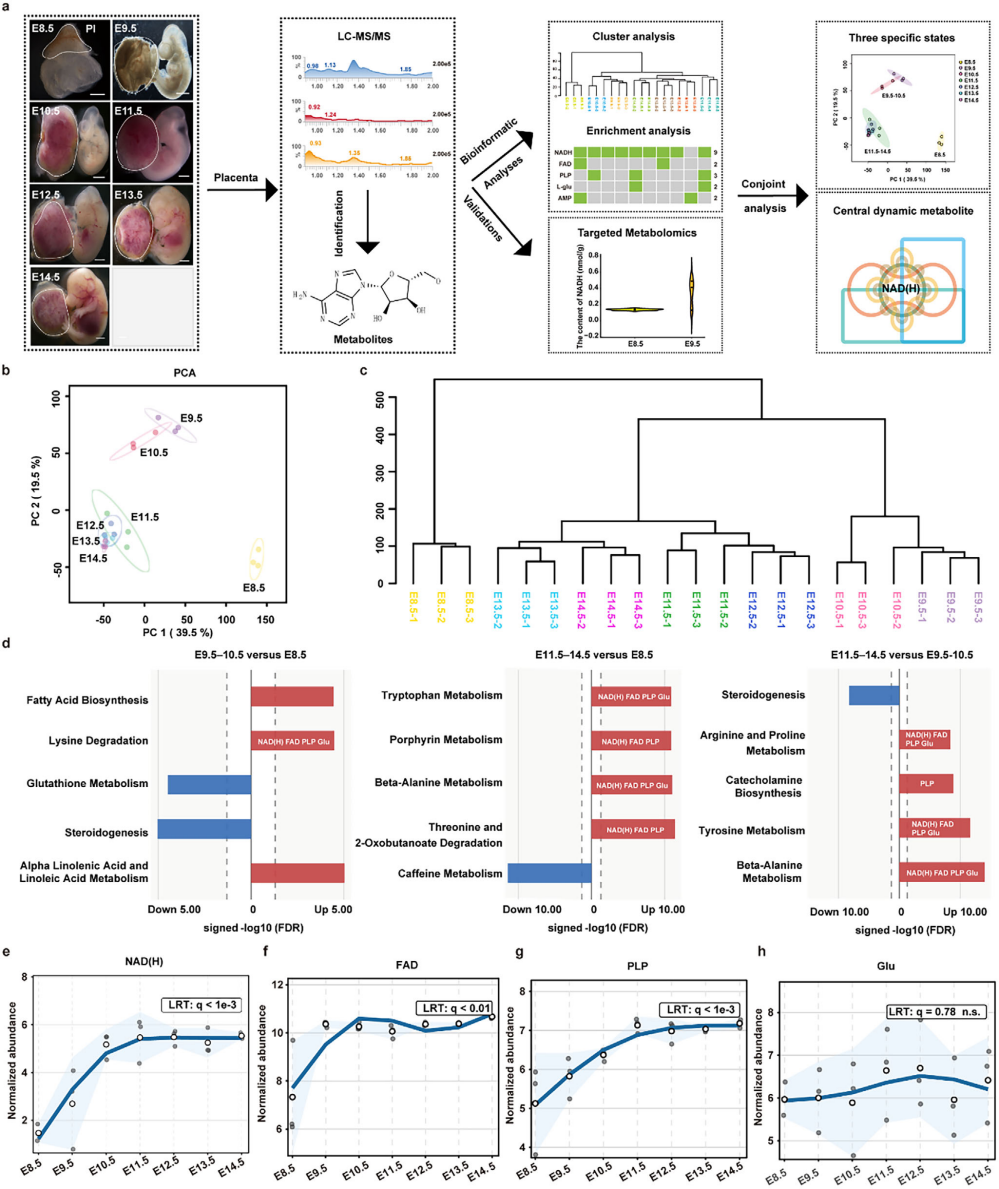
High‐resolution Metabolomics Analysis Reveals Dynamic Metabolic Changes in Mouse Placental Development from E8.5 to E14.5. (a) Schematic diagram of the strategy for sample collection for metabolome profiling in this study. Pl, placenta. Scale bar = 500 µm. (b) Principal component analysis (PCA) plot of stage‐specific samples. The dot color indicates the stage. Samples resolve into three distinct metabolic states—E8.5, E9.5–10.5, and E11.5–14.5—indicating coherent, stage‐dependent separation. (c) The hierarchical clustering dendrogram demonstrates that untargeted metabolomics reliably clusters individual samples based on the stage of the placenta. (d) Pathway enrichment for the indicated contrasts; bars show signed −log10 (FDR) with direction defined by the median change of member metabolites (rightward = up, leftward = down). (e–h) Stage‐resolved trajectories of sentinel metabolites. Normalized abundances of (e) NAD(H), (f) FAD, (g) PLP, and (h) Glu across E8.5–14.5. Points represent samples. Each E8.5 sample contained 27–30 placentas from three litters, and each‐ E9.5‐14.5 sample contained 9 placentas collected from three litters (please see details in Table S1, here and after). q values were adjusted using the Benjamini–Hochberg method from a likelihood‐ratio test across stages.

To characterize the dynamic metabolic features during placentogenesis, we collected 247 E8.5–14.5 placenta tissues from 63 mice (Table S1). LC‐MS/MS was used to perform untargeted metabolomics analysis on these samples (Figure 1a). After data quality control and normalization (see details in Methods), we obtained 4,366 metabolites in total from E8.5–14.5 placentas (Table S2). The number of identified metabolites increased dramatically from 2,721 in E8.5 placentas to 4,140 in E9.5 placentas, and then remained relatively stable during E9.5‐14.5 (Figure S2a).

To holistically visualize the metabolite changes across stages, we performed principal component analysis (PCA) on the detected metabolites. First, samples from each developmental stage were clustered together in the PCA plot, suggesting a high similarity in metabolic features from the same stage and supporting the robustness of our metabolomics results (Figure 1b; Figure S2b). Second, all samples could be categorized into three metabolic states: E8.5, E9.5–10.5, and E11.5–14.5 (Figure 1b), which was consistent with results of hierarchical clustering analysis (Figure 1c), pinpointing the transition of metabolic features from E8.5 to E9.5 and from E10.5 to E11.5. The transitions of metabolic features were consistent with the histological features, as suggested by HE and IF staining results (Figure S2c). At E8.5, even though the initial fusion of the chorioallantoic placenta can be observed, the maternal vessels, fetal vessels, placental labyrinth, and placental syncytiotrophoblasts have not yet formed, indicating that the embryo's growth is still primarily sustained by histiotrophic nutrition. By E9.5–10.5, with the onset of placental labyrinth formation, the fetal vessels (containing nucleated erythroblasts) can be identified in the labyrinth. The maternal blood (enucleated erythrocytes) also begins to perfuse into the labyrinth. The placental syncytiotrophoblasts begin the construction of the maternal‐fetal interface. Compared with the E8.5 placenta, these dramatic morphological changes signify the initiation of maternal‐fetal blood exchange, consistent with the initial transition from histiotrophic to hemotrophic nutrition. From E11.5 onward, compared with the E9.5‐10.5 placenta, the structures of the placental labyrinth and the fetal vascular network both become more complex. The maternal blood perfusion also becomes more affluent. All of these features indicate the enhancement of the maternal‐fetal interface for substrate exchange, which might lead to the complete transition from histiotrophic to hemotrophic nutrition after E11.5.

To reveal the different metabolic pathways activated in the three stage‐specific states, we performed pathway enrichment analysis on all detected metabolites based on the Small Molecule Pathway Database (SMPDB). Compared with E8.5, metabolic pathways like fatty acid biosynthesis, α‐linolenic/linoleic acid metabolism, and lysine degradation were enriched and upregulated in E9.5–10.5 (Figure 1d, left; Table S3). In E11.5–14.5 versus E8.5, enrichment pathway shifted toward tryptophan/porphyrin/β‐alanine metabolism, threonine and 2‐Oxobutanoate degradation (Figure 1d, middle; Table S3). These pathways are compatible with mid‐gestation requirements for oxygen handling and electron transfer (porphyrin) and for immune/endocrine interfacing at the maternal–fetal boundary (tryptophan derivatives) [18, 19]. Placental tryptophan metabolism encompasses serotonin and kynurenine branches implicated in vascular regulation, immune tolerance, and fetal neurodevelopment, while porphyrin biosynthesis supports hemoproteins/cytochromes needed for oxygen transport and mitochondrial respiration as the exchange interface matures [19, 20]. In E11.5–14.5 versus E9.5–10.5, enrichment analysis further revealed upregulation of arginine and proline/tyrosine/β‐alanine metabolism, and catecholamine biosynthesis pathways, while steroidogenesis was comparatively downregulated (Figure 1d, right, Table S3). The L‐arginine–nitric oxide pathway is a key regulator of feto‐placental vascular tone and arterial remodeling, and pharmacologic inhibition or genetic disruption of nitric oxide synthase has been shown to impair uterine–placental perfusion [21]. These shifts align with the denser laminin network within the placental labyrinth and remodeling of proliferin signals in the junctional zone (Figure S2c), indicating evolving transport capacity and junctional architecture during mid‐gestation [10]. Across the many enriched pathways, several metabolic cofactors emerged as top differentially abundant metabolites (DAMs), notably FAD, NAD(H), and pyridoxal‐5’‐phosphate (PLP), as well as Glu (Figure 1d). Relative to E8.5, these DAMs were consistently upregulated at E9.5–14.5 (Figure S2d), and longitudinal measurements corroborated stage‐associated increases in NAD(H), FAD, PLP, and Glu, with NAD(H) showing the most drastic change, and Glu showing the weakest trend amongst them (Figure 1e–h).

**FIGURE 2:**
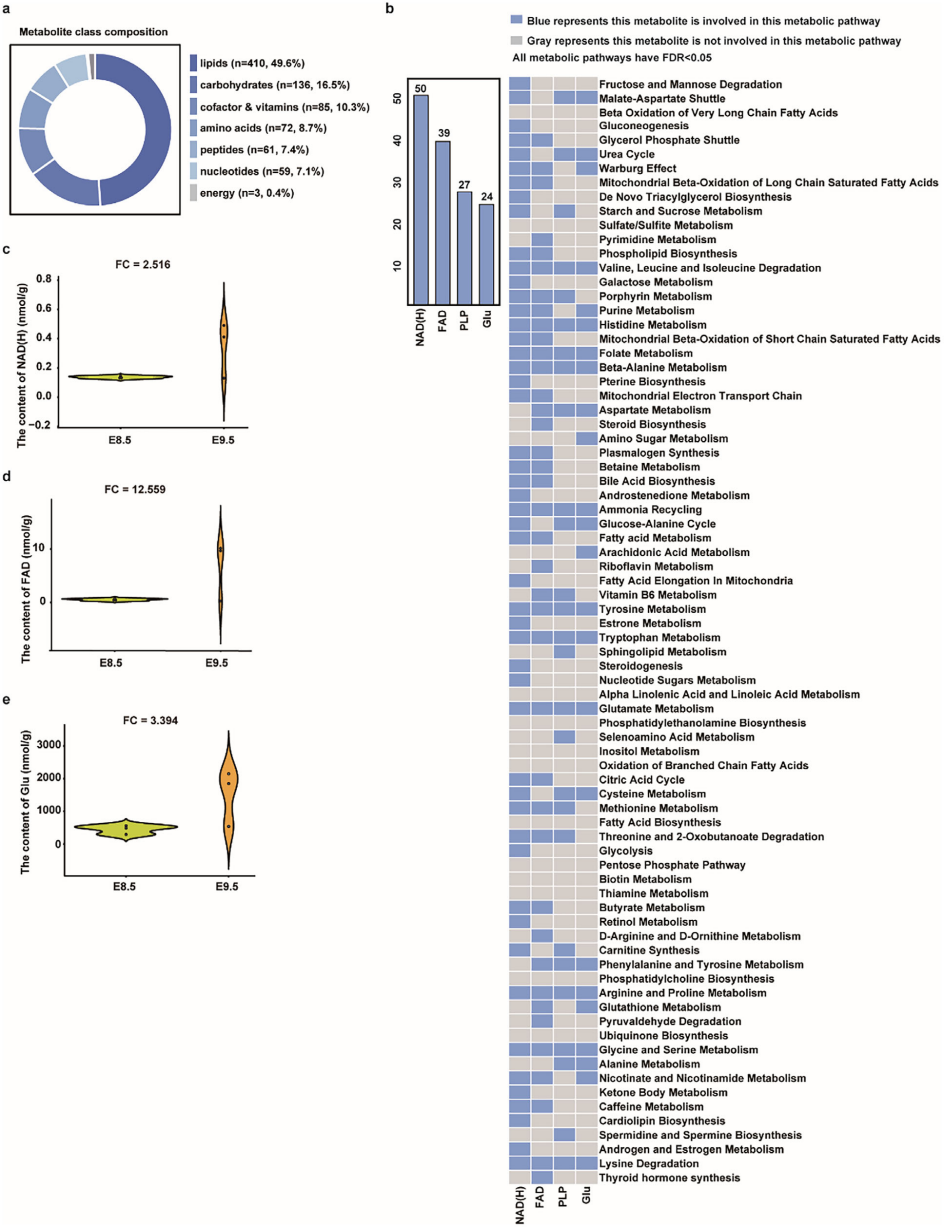
The Differential Metabolite Features of E8.5–9.5 Placentogenesis. (a) Global metabolite class composition from untargeted LC–MS/MS analysis, showing the proportion of annotated features by KEGG class (lipids, carbohydrates, cofactors & vitamins, amino acids, peptides, nucleotides, energy). (b) Bar chart and heatmap show the number and distribution of NAD(H), FAD, PLP, and Glu involved in metabolic pathways in E8.5‐9.5. Gray represents that the metabolite is not involved in this metabolic pathway; blue indicates that this metabolite is involved in this metabolic pathway. (c–e) Quantification of (c) NAD(H), (d) FAD, and (e) Glu levels at E8.5 and E9.5 by targeted metabolomics detection (units: nmol g^−^^1^ tissue); numbers indicate fold change (FC) from E8.5 to E9.5. Points represent samples. Each E8.5 sample contained 27–30 placentas from three litters, and each E9.5‐14.5 sample contained 9 placentas collected from three litters (please see details in Table S1).

Next, we investigated the first metabolites transition from E8.5 to E9.5 in more detail by untargeted metabolomics analysis. In this period, DAMs were dominated by lipids (49.6%), with additional contributions from carbohydrates (16.5%) and from vitamins and cofactors (10.3%), etc., pointing to early lipid remodeling coupled to cofactor chemistry (Figure 2a; Table S3). NAD(H) was the most pathway‐associated DAM (50 pathways), primarily engaged in redox metabolism (e.g., fructose/mannose degradation, malate‐aspartate shuttle, gluconeogenesis) between E8.5 and E9.5 (Figure 2b; Table S3). Targeted metabolomics detection further confirmed that NAD(H), FAD, and Glu were increased in placentas from E8.5 to E9.5 (Figure 2c–e, Figure S3). In addition, the detected metabolites between E10.5 and E11.5 were enriched in pathways related to amino acid metabolism, such as arginine and proline metabolism, and tyrosine metabolism, consistent with previously reported literature [22, 23]. NAD(H) was also involved in these pathways (Table S3).

Taken together, these data define a placental metabolomic atlas for E8.5–14.5 in which metabolic programs align with histological changes. Dramatic metabolomic changes between E8.5, E9.5–10.5, and E11.5–14.5 were explored, and NAD(H) was identified as one of the central placental DAMs.

### 2.2 Transcriptomic Sequencing and Analyses: Verify the Dynamic Metabolic Features During Mouse Placentogenesis

**FIGURE 3:**
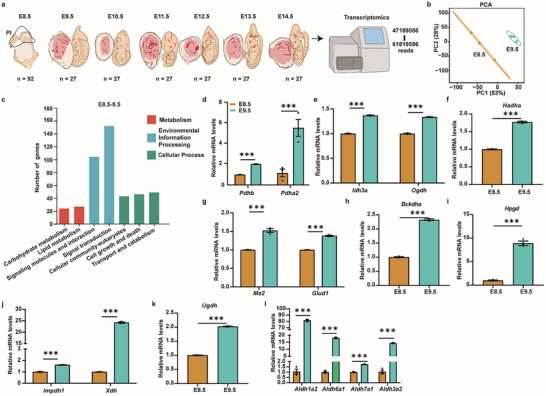
Transcriptomic and Metabolomic Features Reveal Similarities in E8.5–14.5 Placentogenesis. (a) Schematic diagram of the transcriptome analysis for up‐ and down‐regulated genes in E8.5–14.5 placentogenesis. n: number of placentas. (b) PCA of the variance‐stabilized counts shows clear separation between E8.5 and E9.5 placentas (PC1 and PC2 explain 53% and 28% of variance, respectively). Points represent samples. Each E8.5 sample contained 29–31 placentas from three litters, and each E9.5–14.5 sample contained 9 placentas collected from three litters (please see details in Table S1). (c) Pathway category distribution of differentially expressed genes (DEGs) between E9.5 and E8.5 (bar heights represent the number of genes; KEGG super‐categories color‐coded). (d‐l) qRT‐PCR validation of the levels of representative enzymes involved in redox metabolism, comparing E9.5 (green) to E8.5 (orange): (d) *Pdhb, Pdha2*, (e) Idh3a, *Ogdh*, (f) *Hadha*, (g) *Me2, Glud1*, (h) *Bckdha*, (i) *Hpgd*, (j) *Impdh1, Xdh*, (k) *Ugdh*, (l) *Aldh1a2, Aldh6a1, Aldh7a1, Aldh3a2*. Bars show mean ± s.e.m. n = 3. Statistics: two‐tailed unpaired Student's *t*‐test; ^***^: *p* < 0.001.

To explore the regulatory mechanisms underlying the dynamic metabolic changes during placentogenesis, we further collected 254 mouse placentas for bulk transcriptomic sequencing (Figure 3a; Tables S1 and S4). The reads of cDNA libraries ranged from 47,189,056 to 61,819,596 (Table S1). After quality control, 99.04%−99.23% of reads were retained (Table S1). Results from PCA and correlation analyses revealed that biological replicates of each developmental stage clustered closely together. Furthermore, all placenta samples could be classified into three stage‐specific clusters: E8.5, E9.5–10.5, and E11.5–14.5, which is consistent with the histological and metabolomics findings (Figures S2c and S4a, b; Figure 1b, c). Compared with E8.5 transcripts, the number of differentially expressed genes (DEGs) increased from 1194 at E9.5 to 7359 at E14.5 (Figure S4c), suggesting that the placenta undergoes progressive, stage‐dependent transcriptional changes from E9.5 to E14.5 [7]. At stages E8.5 to E9.5, the KEGG annotation of metabolism‐associated genes is predominantly enriched for pathways related to lipid and carbohydrate metabolism (Figure 3c), a pattern that is consistent with the major metabolite categories of enriched DAMs in the same stage (Figure 2a; Table S3). We have identified NAD(H) as an up‐regulated central DAM in placentogenesis during E8.5–14.5, and an absolute increase from E8.5 to E9.5 (Figures 1e and 2c; Figure S2d). Considering that NAD(H) accumulation results from multiple redox‐associated metabolic pathways (Figure 2b), we therefore profiled gene expression of enzymes involved in these redox pathways. Compared with E8.5, the expression of genes that are involved in the redox regulation of NAD(H), like *Pdhb, Pdha2, Idh3a*, *Ogdh, Hadha, Me2, Glud1, Bckdha, Hpgd*, *Impdh1, Xdh, Ugdh, Aldh1a2, Aldh6a1, Aldh7a1*, and *Aldh3a2* is increased in E9.5 (Figure 3d‐l). In addition, *Rfk* and *Flad1* were upregulated, supporting an increase in FAD during E8.5–9.5 (Figures S2d and S4d, e; Figure 1f).

Taken together, combined with the transcriptome analyses for mouse E8.5–14.5 placentas, we found consistent dynamic patterns in metabolomic and transcriptomic changes during placentogenesis, and revealed the potential mechanisms for NAD(H) accumulation in the enriched metabolic pathways.

### 2.3 NAD(H) and Glu Promote the Body Length Extension of the IVC Embryos

**FIGURE 4:**
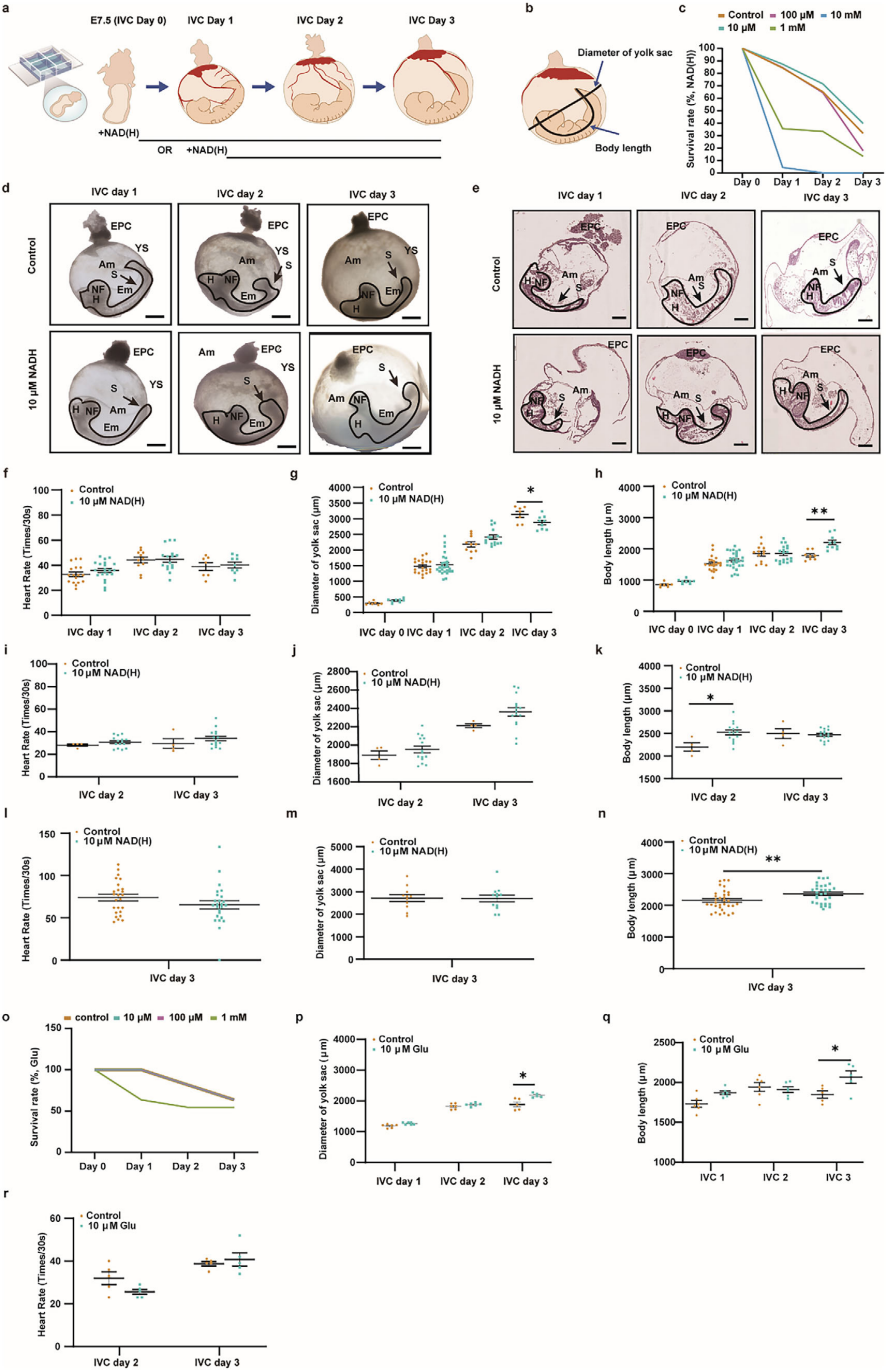
NAD(H) Promotes Body Length Extension in IVC Embryo. (a) Schematic overview of the mouse embryo in vitro culture system. (b) Illustration of body length and yolk sac diameter measurements. (c) Survival curves of IVC embryos after NAD(H) administration. (n = 63). (d) Brightfield images of mouse embryos on IVC day 1–3. Scale bar = 500 µm. EPC, ectoplacental cone. YS, yolk sac. H, heart. Em, embryo. NF, neural fold. Am, amnion. S, somite. The solid line indicates the embryo. (e) H&E staining images of mouse embryos on IVC day 1–3. Scale bar = 500 µm. EPC, ectoplacental cone. YS, yolk sac. H, heart. NF, neural fold. Am, amnion. S, somite. The solid line indicates the embryo. (f) Effects of NAD(H) on the heart rate of IVC embryos. Data are mean ± s.e.m. n (control) = 17, n (NAD(H)) = 21. (g) Effects of NAD(H) on the diameter of the yolk sac in IVC embryos. Data are mean ± s.e.m. n (control) = 20, n (NAD(H)) = 26, Statistics: two‐tailed unpaired Student's *t*‐test, ^*^: *p* ≤ 0.05. (h) Effects of NAD(H) on the body length of IVC embryos. Data are mean ± s.e.m. n (control) = 22, n (NAD(H)) = 28, Statistics: two‐tailed unpaired Student's *t*‐test, ^**^: *p* ≤ 0.01. (i) Effects of NAD(H) on the heart rate of IVC embryos. Data are mean ± s.e.m. n (control) = 4, n (NAD(H)) = 15. (j) Effects of NAD(H) on the diameter of the yolk sac in IVC embryos. Data are mean ± s.e.m. n (control) = 4, n (NAD(H)) = 15. (k) Effects of NAD(H) on the body length of IVC embryos. Data are mean ± s.e.m. n (control) = 4, n (NAD(H)) = 15, Statistics: two‐tailed unpaired Student's *t*‐test, ^*^: *p* ≤ 0.05. (l) Effects of NAD(H) on the heart rate of IVC embryos using a rotating cultivation system. Data are mean ± s.e.m. n (control) = 24, n (NAD(H)) = 24. (m) Effects of NAD(H) on the diameter of the yolk sac in IVC embryos using a rotating cultivation system. Data are mean ± s.e.m. n (control) = 33, n (NAD(H)) = 30. (n) Effects of NAD(H) on the body length of IVC embryos using a rotating cultivation system. Data are mean ± s.e.m. n (control) = 33, n (NAD(H)) = 30, Statistics: two‐tailed unpaired Student's *t*‐test, ^**^: *p* ≤ 0.01. (o) Survival curves of IVC embryos after Glu administration. n = 6. (p–r) Effects of Glu on the diameter of the yolk sac, body length, and heart rate in IVC embryos. Data are mean ± s.e.m. n = 6. Statistics: two‐tailed unpaired Student's *t*‐test, ^*^: *p* ≤ 0.05.

Recent studies have shown that NAD(H) can be transported across plasma membranes [24, 25, 26]. Thus, the increased NAD(H) levels during placentogenesis from E8.5 to E14.5 led us to ask if the dramatic rise in placental NAD(H) exerts effects on embryonic development. To answer this question, we supplemented in vitro cultured E7.5 embryos with NAD(H) and examined various developmental characteristics, including survival rates, heart rate, body length, and yolk sac diameter (Figure 4a, b; Video S1).

Based on previous reports for NAD(H) working concentrations in vitro [27], we added 10, 100 µm, 1, and 10 mm NAD(H) to the IVC medium from day 0, and found that 10 µm NAD(H) showed the highest embryo survival rate during culture (Figure 4c). Therefore, we used 0 and 10 µm NAD(H) as the control and experimental groups, respectively. Brightfield and histological staining images suggested the heart, somites, and neural tube had developed in both control and experimental IVC Day 1 embryos, and were maintained in IVC Days 2 and 3 (Figure 4d, e). On IVC Days 1 and 2, there were no significant differences between the control and NAD(H) group in heartbeat, diameter of the yolk sac, and body length (Figure 4f–h). On IVC Day 3, there was no significant difference in heart rate between the control and experimental group embryos (Figure 4f). However, the experimental group embryos exhibited shorter yolk sac diameter (control vs. experiment: 3139.2 ± 96.5 µm vs. 2878.4 ± 71.4 µm, *p* = 0.0456, Figure 4g), but longer body length (control vs. experiment: 1789.7 ± 52.6 µm vs. 2203.8 ± 72.8 µm, *p* = 0.0045, Figure 4h). When we added the NAD(H) from IVC Day 1, only body length increased in the experimental group on IVC Day 2 (Figure 4i–k). Further, we employed a rotating culture system to better support embryonic growth and added NAD(H) from IVC Day 0. On IVC Day 3 following rotating culture, there was no significant difference in heart rate and yolk sac diameter between the control and experimental group embryos (Figure 4l, m). However, the experimental group embryos again exhibited longer body length (control vs. experiment: 2155.6 ± 60.0 µm vs. 2366.3 ± 54.1 µm, *p* = 0.0091, Figure 4n).

To investigate the roles of other placental DAMs such as Glu, FAD, and PLP during embryonic development, we supplemented IVC embryos with these metabolites. For Glu‐treated embryos, based on the survival rate, both 10 and 100 µm Glu had comparable effects and supported higher embryo survival than 1 mm during 3 days of IVC. Therefore, we used 0 and 10 µM Glu as the control and experimental groups, respectively (Figure 4o). When Glu was added on IVC Day 0, embryos treated with 10 µm Glu on IVC Day 3 exhibited significantly greater yolk sac diameter (control vs. experiment: 1888.3 ± 82.2 µm vs. 2181.1 ± 31.2 µm, *p* = 0.0103, Figure 4p), and a longer body length (control vs. experiment: 1849.5 ± 44.9 µm vs. 2066.9 ± 77.7 µm, *p* = 0.0417, Figure 4q), indicating that optimal levels of exogenous Glu can also promote embryonic growth. Meanwhile, the heart rate showed no statistically significant differences (Figure 4r), underscoring the importance of placental control of optimal DAMs concentrations. For FAD‐treated embryos, based on the survival rate, both 10 and 100 µM FAD could support similar and higher embryo survival than 1 mM FAD during 3 days of culture. Therefore, we used 0 and 10 µm FAD as the control and experimental groups, respectively, starting on IVC Day 0 (Figure S5a). On IVC Day 3, there was no significant difference in heart rate (Figure S5b), whereas embryos treated with 10 µm FAD exhibited shorter yolk sac diameter and body length (yolk sac diameter: control vs. experiment: 2284.7 ± 35.7 µm vs. 1762 ± 42.9 µm, *p* = 0.00001; body length: control vs. experiment: 2189.5 ± 98.6 µm vs. 1662 ± 31.2 µm, *p* = 0.0034, Figure S5c, d), indicating exogenous FAD reduces embryonic growth. In contrast, PLP supplementation had no significant effects on embryonic growth (Figure S5e–h).

Taken together, these findings demonstrate that placental DAMs exhibit distinct effects on multiple developmental features of in vitro cultured embryos.

### 2.4 NAD(H) Could Accelerate the Segmentation Clock

**FIGURE 5:**
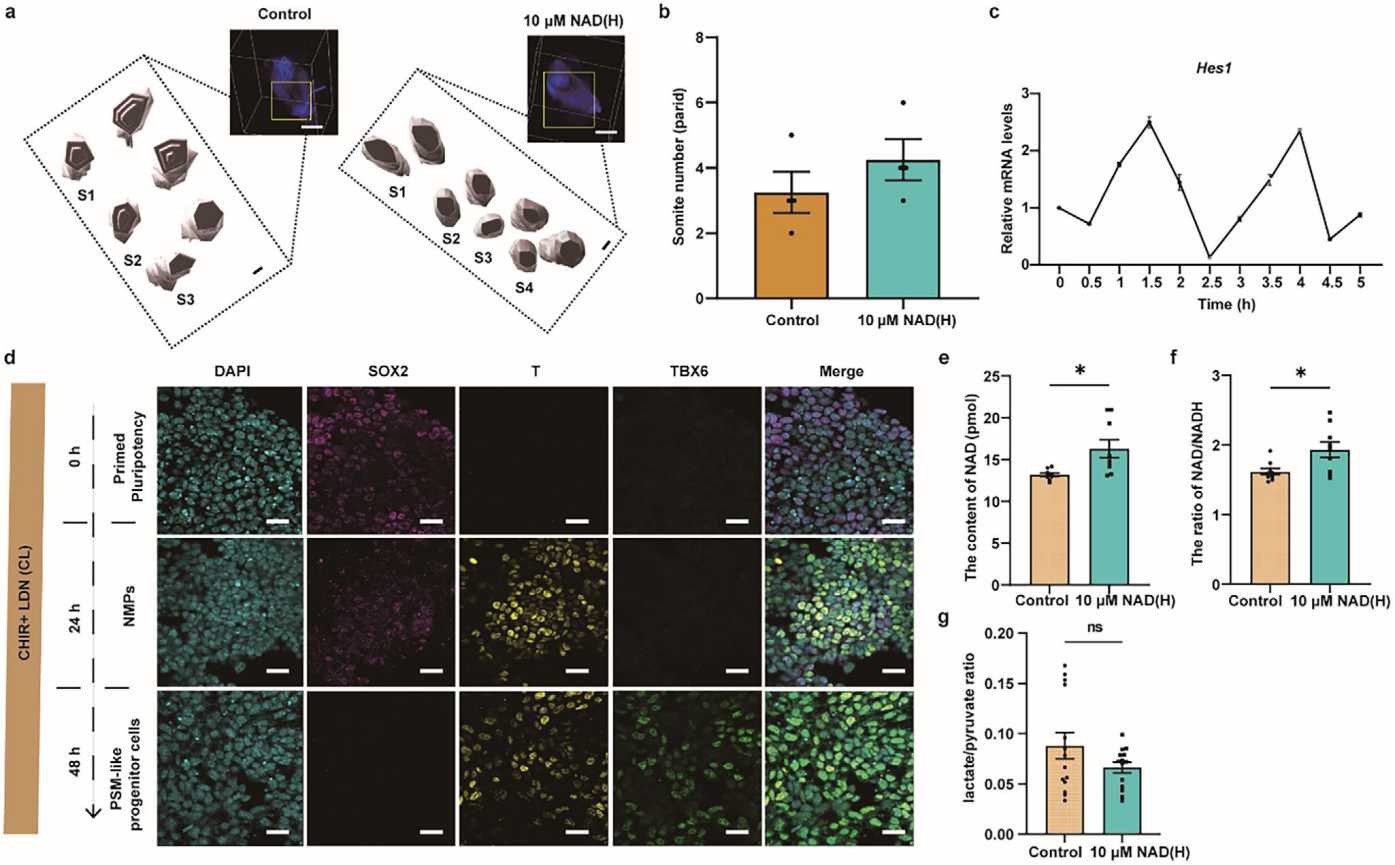
NAD(H) Accelerates the Segmentation Clock. (a) Whole‐mount immunofluorescence and 3D reconstruction showing somite morphology in control and NAD(H)‐treated embryos. S = somite. Scale bar = 50 µm. (b) Quantification of somite numbers per embryo under control and NAD(H)‐treated conditions. (c) qRT‐PCR showing oscillatory expression of *Hes1* mRNA. (d) Immunofluorescence of the pluripotency factor SOX2 and the PSM‐like cell markers T and TBX6. Scale bar = 50 µm. NMPs, neuromesodermal progenitors. (e–g) Quantification of (e) NAD, (f) Whole‐cell NAD(H) ratio, (g) Extracellular lactate/pyruvate ratio in PSM‐like progenitor cells after NAD(H) treatment. Data are mean ± s.e.m. n = 13, Statistics: two‐tailed unpaired Student's *t*‐test, ^*^: *p* ≤ 0.05, ns: no significance.

Considering that the relationship between Glu and ESCs or embryonic development has been extensively studied elsewhere [28, 29, 30, 31, 32], this work focused on the role of NAD(H) in embryonic and body length development. The somites are a set of bilaterally paired blocks along the anterior‐posterior axis in segmented animals derived from paraxial mesoderm during early embryonic development. Somites consist of groups of epithelial cells that assemble as rosettes, whose number is closely related to the length of the embryonic body. Motivated by the body‐length gain observed in IVC embryos treated with 10 µm NAD(H) (Figure 4f–n), we asked whether somite formation was affected. To verify this hypothesis, we compared the number of somites between IVC embryos from control and experimental groups using whole‐mount staining with phalloidin and DAPI (Video S2). It turned out that NAD(H)‐treated embryos tended to displayed a higher mean somite number than control embryos on average (control vs. experiment: 3.25 ± 0.63 vs. 4.25 ± 0.63 pairs, n = 4, *p* = 0.30399, Figure 5a, b).

Considering that somites are generated at fixed temporal intervals, i.e., driven by the segmentation clock [33, 34], we hypothesized that the addition of NAD(H) might quicken the segmentation clock. To identify the effect of NAD(H) on the segmentation clock, we established and characterized mESC‐derived PSM‐like progenitor cells based on the periodic oscillation of the cycling gene *Hes1* and the co‐expression of T and TBX6 (Figure 5c, d; Figure S6a–c). It has been reported that the mitochondrial NAD(H) redox ratio can modulate segmentation clock dynamics [35]. We therefore first measured the levels of whole‐cell NAD and the whole‐cell NAD(H) redox ratio in PSM‐like progenitor cells with or without NAD(H) treatment (experiment or control group, respectively). The results indicated that compared with the control group, the experimental group showed increased whole‐cell NAD levels (control vs. experiment: 13.2 ± 0.2 pmol vs. 16.3 ± 1.1 pmol, *p* = 0.0119) and the NAD(H) redox ratio (control vs. experiment: 1.6 ± 0.1 vs. 1.9 ± 0.1, *p* = 0.0197) (Figure 5e, f). Next, we measured the pyruvate/lactate ratio, a proxy for the cytosolic NAD(H) redox ratio [36]. We found there was no significant difference in the pyruvate/lactate ratio between control and experimental groups (control vs. experiment: 0.09 ± 0.01 vs. 0.06 ± 0.01, *p* = 0.1368), suggesting no changes in the cytosolic NAD(H) redox ratio (Figure 5g), so that the changes in whole‐cell NAD(H) redox ratio may be from mitochondria.

Taken together, these data indicate that NAD(H) accelerates the segmentation clock, increases the number of somites, and thus promotes embryonic body length extension by elevating the mitochondrial NAD(H) redox ratio, a known regulator of the segmentation clock [35, 37, 38, 39].

### 2.5 NAD(H) Enhances the Proliferation of PSM‐Like Progenitor Cells

**FIGURE 6:**
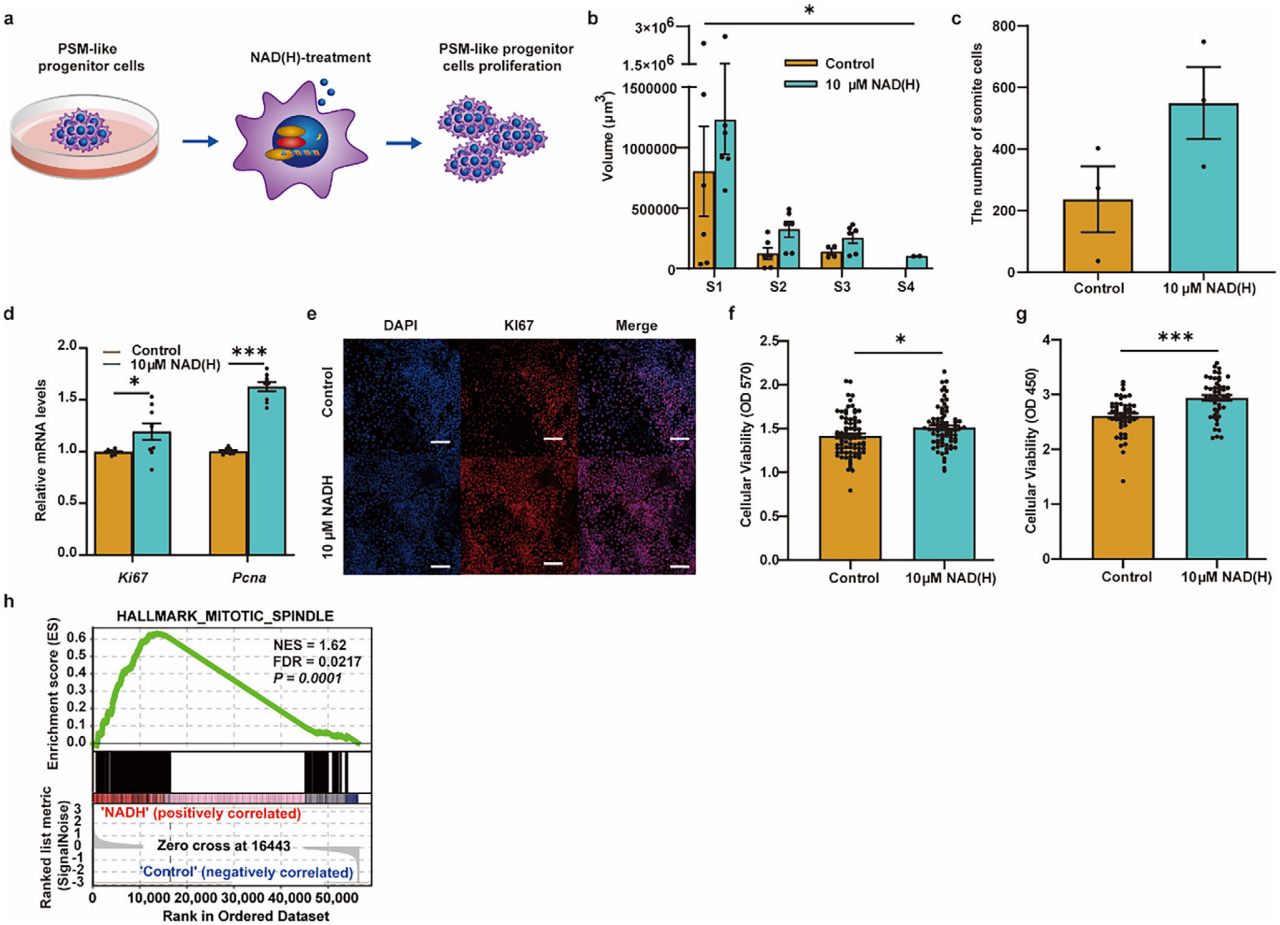
NAD(H) Enhances the Proliferative Capacity of PSM‐like Progenitor Cells. (a) Schematic illustration of the proposed mechanism by which NAD(H) promotes proliferation of PSM‐like progenitor cells. (b) The somite volumes of IVC embryos. Data are mean ± s.e.m. n =  4. S: somite. Statistics: Kruskal–Wallis test, ^*^: *p* < 0.05. (c) Average cell number per somite in IVC embryos. Data are mean ± s.e.m. n  =  4. (d) qRT‐PCR results show that NAD(H) promotes the transcriptional level of PSM‐like cell proliferation markers *Ki67* and *Pcna*. Data are mean ± s.e.m. n = 3, Statistics: two‐tailed unpaired Student's *t*‐test, ^*^: *p* ≤ 0.05, ^***^: *p* ≤ 0.001. (e) Immunofluorescence staining of proliferation markers KI67 in control and NAD(H)‐treated PSM‐like cells. Scale bar = 50 µm. (f, g) Cell viability assays using (f) CCK‐8 and (g) MTT show that NAD(H) enhances the proliferative activity of PSM‐like progenitor cells. Data are mean ± s.e.m. n (CCK‐8) = 49, n (MTT) = 78, Statistics: two‐tailed unpaired Student's *t*‐test, ^*^: *p* ≤ 0.05, ^***^: *p* ≤ 0.001. (h) Pre‐ranked GSEA of RNA‐seq identifies enrichment of HALLMARK_MITOTIC_SPINDLE in the NAD(H)‐treated group. NES = 1.62, FDR = 0.0217, *p* = 0.0001.

The length of the embryonic body is also closely related to the somite volumes resulting from the proliferation of PSM‐like progenitor cells [40]. To verify whether NAD(H) increases somite volume, we performed 3D image reconstructions of somites in IVC embryos from control and experimental groups. We found that somite volume in the experimental groups exhibited an increase compared to controls (*p* = 0.0219, Figure 6a, b). Similarly, the average number of cells per somite showed a trend toward an increase in the experimental group compared to controls (control vs. experiment: 237 ± 107 vs. 550 ± 117, *p* = 0.12, Figure 6c), indicating that NAD(H) may promote the proliferation of PSM‐like progenitor cells.

To determine if NAD(H) proliferation enhances PSM‐like progenitor cells, we checked the expression of proliferation markers like *Pcna* and *Ki67* in the PSM‐like progenitor cells from control and experimental groups. The qRT‐PCR and IF results showed that NAD(H) significantly upregulated the transcriptional and protein expression levels of *Pcna* and *Ki67* in PSM‐like progenitor cells (Figure 6d, e). CCK‐8 and MTT analyses further confirmed the significant enhancement of proliferative capacity in PSM‐like progenitor cells treated with NAD(H) (Figure 6f, g). These results indicate that NAD(H) could promote PSM‐like progenitor cells proliferation, which resulted in the extension of the embryonic body length. Next, we performed RNA‐seq on both PSM‐like progenitor cells and NAD(H) treated PSM‐like progenitor cells, and we observed that DEGs were enriched in the mitotic spindle, G2/M checkpoint, PI3K–AKT–MTOR, and TGF‐β signaling modules, further supporting the hypothesis that NAD(H) promotes PSM‐like progenitor cells’ proliferation (Figure 6h; Figure S7a–c).

Taken together, these findings support an association between NAD(H) supply and enhanced proliferative capacity in PSM‐like progenitor cells.

## 3 Discussion

Our understanding of mouse placenta metabolism during placentogenesis is limited due to the lack of reliable datasets. In this study, we present a stage‐based histology‐metabolome–transcriptome atlas of mouse placentas (E8.5–14.5) that reveals three distinct metabolic states (E8.5, E9.5–10.5, E11.5–14.5). This unsupervised analysis‐based temporal partitioning of the placental metabolome is consistent with the histological changes, marked by the appearance of the placental labyrinth from E8.5 to E9.5, and the increase in fetal and maternal blood perfusion in the labyrinth from E10.5 to E11.5, accompanied by a transition in metabolism from histiotrophic to hemotrophic nutrition. The metabolic features between E8.5 and 9.5 show a coordinated shift from lipid and carbon metabolism toward oxygen handling and electron transfer, mainly related to the dynamic changes in cofactors like NAD(H), FAD, and PLP.

To identify the effects of NAD(H) on embryonic development, IVC embryos were treated with NAD(H), which exhibited increased body length. Even though the development of IVC embryos does not perfectly recapitulate that of in vivo embryos, preventing the system from mimicking later stages of embryogenesis after IVC Day 3, the IVC embryos can effectively recapitulate the key developmental events of interest, such as heart formation, yolk sac expansion, and somite development. NAD(H) showed no effects on yolk sac diameter and heart rate. Notably, the body length, yolk sac diameter, and heart rate are separately regulated by different factors. For example, body elongation is closely related to the segmentation oscillation clock and somite volumes [41]. The yolk sac diameter expansion relies on the proliferation of yolk sac endoderm and mesoderm [42]. Our experiments indicate that yolk sac diameter is also regulated by optimal FAD and Glu levels. The heart rate is influenced by the regulation of ion channels, the supply of ATP [32], and the maturity of cardiomyocytes. While our experiments did not reveal any placental DAMs that regulate embryonic heart rate, other groups have found that the lactate to fatty acid ratio can regulate cardiomyocyte maturation and cardiac development [43, 44]. Thus, different organs’ development relies on different metabolic pathways for regulation.

The trio of NAD, NADH, and the NAD(H) redox ratio represents different facets of the same central metabolic system. The current studies mainly focus on NAD or NAD(H) redox ratio‐related metabolic pathways; however, NAD(H) itself is the critical factor that determines both. The function of NAD(H) in physiological and pathological human placentogenesis remains fragmentarily reported. For physiological pregnancy, pathways related to NAD metabolism are upregulated in the second trimester compared to the first trimester placentas [45]. For pathological pregnancy, in preeclampsia and placental insufficiency, placental tissues exhibit impaired electron transport chain activity accompanied by an aberrant NAD(H) redox ratio, which triggers placental dysfunction [46, 47]. Notably, NAD‐dependent cortisol oxidation is approximately threefold higher in placental tissues derived from pregnancies at high risk for preeclampsia compared to those from normal pregnancies [48]. In gestational diabetes mellitus (GDM), the fetal and placental endothelial compartments both display reduced NAD levels coupled with diminished Sirtuin (SIRT1/SIRT3) activity, which is closely associated with redox‐linked metabolic stress in GDM [49]. Sustained redox stress signaling in fetal endothelial cells in GDM compromises the cellular defenses against oxidative stress in utero, thereby predisposing offspring to an increased risk of type 2 diabetes mellitus and cardiovascular diseases in later life [50]. These metabolic abnormalities augment oxidative stress and induce placental mitochondrial damage, resulting in fetal growth restriction (FGR), with all these changes consistently linked to reduced NAD levels [51]. Collectively, these findings highlight that NAD(H) and its redox homeostasis are indispensable for normal placental development and function, indicating NAD(H)‐related pathways could be promising diagnostic biomarkers and potential therapeutic targets for pregnancy‐related disorders.

Further experiments in placental organoids and animal models are warranted. For example, placental organoids can be used to test how endogenous DAMs regulate trophoblast proliferation and differentiation. A maternal–fetal, dual‐channel placenta‐on‐a‐chip incorporating organoid‐derived monolayers can be used to quantify trans‐barrier transport of DAMs and assess barrier integrity, thereby linking the effects of placental substrate supply to embryonic organoid developmental phenotypes. Using placental organoids to model pathologies such as preeclampsia, we can evaluate the mechanistic contribution of DAMs to disease pathophysiology in the future. Taken together, our findings provide a metabolic perspective for understanding placentogenesis and its effects on embryonic developmental biology.

Some limitations in this study should be clarified: our litter‐level pooling strategy improved analytical robustness and sensitivity to stage‐to‐stage differences, but it may mask placenta‐to‐placenta variability and preclude sex‐stratified analyses. Future studies will analyze male and female placentas separately without pooling where feasible.

## 4 Experimental Section

### 4.1 Animals and Placenta Samples

All animal procedures complied with ethical standards approved by the Institute of Zoology, Chinese Academy of Sciences (Approval No. IOZ‐IACUC‐2021‐176). Healthy ICR mice from SPF (Beijing) Biotechnology were kept in controlled conditions, with a 12 h light/dark cycle and stable temperature and humidity, while having free access to food and water. For mating, male and female mice aged 6–8 weeks were paired at a male‐to‐female ratio of 2:3 overnight. Detection of a vaginal plug marked E0.5. Placental samples from E8.5 to E14.5 were collected after cervical dislocation euthanasia. At each stage, placentas were obtained from nine pregnant dams, representing nine independent litters. Based on previous reports [52, 53], for metabolomics and transcriptomics, placentas from three litters were pooled to generate one litter‐level replicate, and three pooled replicates were analyzed per stage. Whole placentas (membranes removed) were used for subsequent experiments.

### 4.2 Untargeted Metabolomic Analysis

Metabolite extraction followed a modified protocol from Shyh‐Chang et al. [54]. Briefly, frozen tissues were mixed 1:1 with pre‐chilled 80% methanol–water (−78°C) and homogenized under liquid nitrogen using a TissuePrep 24 bead mill (Gering, Beijing, China) with stainless‐steel beads (100 Hz, 1–5 min). Lysates were clarified (5 000 ×g, 15 min, 4°C), and supernatants were transferred to LC–MS vials. Chromatography was performed on an Acquity I‐Class UPLC (Waters, USA) with an HSS T3 column (2.1 × 100 mm, 1.7 µm) at 0.4 mL min^−^^1^ using mobile phase A (0.1% formic acid in water) and B (0.1% formic acid in acetonitrile). The gradient was: 0–8.5 min, 95%→50% A; 8.5–12 min, 50%→2% A; 12–16 min, re‐equilibrate at 95% A. Injection volume was 2 µL. Mass spectrometry used a Xevo G2‐XS Q‐TOF (Waters) with ESI in positive and negative modes and UPLC/MSE acquisition. Source parameters were: capillary voltage, +2.5 kV/−2.0 kV; source temperature, 120°C; desolvation temperature, 200°C; cone gas, 30 L h^−^^1^; desolvation gas, 800 L h^−^^1^ (N_2_). Leucine‐enkephalin (5 ng mL^−^^1^) served as the lockmass (m/z 556.2771 in positive mode; m/z 554.2615 in negative mode). MSE used low‐energy 6 eV and a ramped high‐energy 10–40 eV over m/z 50–1200 at 0.2 s scan^−^^1^.

Raw files were processed in Progenesis QI (Nonlinear Dynamics, Waters). Features were aligned across runs, peak picked, and deconvoluted using default centroiding. Putative metabolite identities were assigned by accurate mass, isotopic pattern, and MS/MS fragment matching against HMDB 5.0; only features with consistent retention time across samples and acceptable identification scores were retained for downstream analyses.

For each time point, feature tables were imported to R and harmonized by HMDB identifiers. Table orientation was auto‐resolved, non‐informative columns were removed, and zeros/missing values were imputed as half of the feature‐wise minimum positive intensity to satisfy variance‐stabilizing assumptions. Time‐point matrices were then merged into a single feature‐by‐sample matrix. To mitigate mean–variance dependency, we applied variance stabilization (vsn::vsn2). Residual technical batch effects were removed with limma::removeBatchEffect using the batch factor at the file level and a design that included biological group (E8.5–14.5), preserving biological signal. Biological replicates with identical group labels across batches were summarized by the median for robustness. This batch‐corrected, VSN‐scaled matrix served as the input for all unsupervised analyses.

PCA was performed on centered VSN values from the batch‐corrected matrix. Score plots display 95% data ellipses per developmental stage (E8.5–14.5). Between‐stage similarity was evaluated by a Spearman correlation heatmap computed on per‐stage means (log‐scale VSN). Dendrograms were generated using Euclidean distance and complete linkage; figures maintain developmental ordering to emphasize temporal continuity. To quantify temporal changes, pairwise comparisons were performed for each later stage vs. E8.5 in MetaboAnalyst 5.0 (Statistical Analysis module). For each comparison, data preprocessing used sum normalization, log_2_ transformation, and Pareto or autoscaling as specified. Metabolites with |log_2_ fold‐change| ≥1 and FDR ≤ 0.05 (two‐sided) were defined as differentially abundant metabolites (DAMs). Volcano plots, PCA score plots, and clustering of significant features were produced within MetaboAnalyst.

Functional interpretation used MetaboAnalyst's Metabolite Set Enrichment Analysis (MSEA; Quantitative Enrichment /globaltest) with SMPDB libraries. The background/reference set comprised all detected metabolites that could be mapped to HMDB in our dataset (overall mapping rate: 82%). Pathway directionality (up‐ or down‐regulated) was assigned by the sign of the median normalized abundance change across pathway members in each comparison; mixed signatures were reported as bidirectional/neutral. Enrichment results were summarized as dot plots and pathway overviews.

Metabolite classes used in summary figures were assigned using KEGG compound/class annotations mapped from HMDB identifiers. Where multiple categories were plausible, a prioritized single label was retained to ensure consistency across time points.

To test continuous temporal trends across E8.5–14.5, we fitted for each metabolite a model of normalized abundance as a function of developmental time (numeric), using a natural spline (df = 3) in the full model and an intercept‐only reduced model. Models were compared by analysis‐of‐variance likelihood‐ratio tests, and *p*‐values were adjusted across metabolites using the Benjamini–Hochberg procedure. Fitted curves with 95% confidence intervals were reported for representative metabolites and for selected redox‐relevant species (e.g., NAD(H), PLP, FAD, Glu).

### 4.3 Targeted Metabolomic Analysis

HPLC‐grade methanol, acetonitrile, and chloroform were obtained from Thermo Fisher Scientific (Fair Lawn, NJ, USA). Nucleotide standards (Sigma–Aldrich, St. Louis, MO, USA) were dissolved in 80% methanol/water (v/v) to prepare a 5.0 mg/mL stock solution, followed by serial dilution to generate calibration curves.

Frozen tissue samples (10 mg) were thawed on ice, homogenized with 20 µL deionized water and 10 grinding beads (BB24, Next Advance Inc., Troy, NY, USA) using a two‐step homogenization protocol (3 min each step). After initial homogenization, 400 µL of ice‐cold 80% methanol/water (v/v) was added for secondary homogenization. The homogenate was centrifuged at 18,000 × g for 20 min at 4°C (Microfuge 20R, Beckman Coulter, Brea, CA, USA), and 60 µL of supernatant was collected for LC‐MS analysis.

Analyses were performed on an ACQUITY UPLC‐Xevo TQ‐S system (Waters Corp., Milford, MA, USA). Chromatographic separation was achieved using an AdvanceBio MS Spent Media column (2.7 µm, 2.1 × 100 mm) maintained at 35°C. Mobile phases consisted of: (A) 15 mm ammonium acetate in water‐acetonitrile (85:15, v/v) and (B) 15 mm ammonium acetate in water‐acetonitrile (20:80, v/v). The gradient elution program was as follows: 0–0.5 min (80% B), 0.5‐6 min (linear decrease to 40% B), 6–7.2 min (40% B), 7.2–7.5 min (linear increase to 80% B), 7.5–9 min (80% B) at a flow rate of 0.30 mL/min. Injection volume: 2 µL. Mass spectrometry parameters: ESI+ ionization mode; capillary voltage 3 kV; source temperature 150°C; desolvation temperature 500°C; desolvation gas flow 1000 L/h.

### 4.4 Transcriptome Analysis

Total RNA was extracted with TRIzol Reagent (Invitrogen, 15596018) following the manufacturer’s instructions. RNA integrity was confirmed (RIN ≥7.0) prior to library preparation. Strand‐specific mRNA libraries were prepared by Shanghai Majorbio Bio‐pharm Technology Co., Ltd. using the Illumina Stranded mRNA Prep kit from 1 µg input. Polyadenylated transcripts were enriched with oligo(dT) beads, fragmented, and reverse‐transcribed using random hexamers and SuperScript reagents; ∼300‐bp inserts were size‐selected and PCR‐enriched. Libraries were sequenced on an Illumina NovaSeq 6000 to generate paired‐end 2 × 150 bp reads. Adapters and low‐quality bases were removed with SeqPrep (minimum overlap ≥15 bp; 3′ Phred trimming enabled). Clean reads were aligned to the mouse reference genome GRCm38 using HISAT2 (v2.x) with strand‐specific settings appropriate for dUTP‐based libraries (–rna‐strandness RF), maximum intron length 500 kb, and default splice‐aware parameters. Mapping metrics (overall/unique rates, insert‐size distribution, 5′→3′ coverage) were compiled for QC. Gene‐level abundance was estimated with RSEM (v1.x) against the GRCm38 transcriptome to obtain expected counts and FPKM/TPM. Raw expected counts were used for statistical testing; FPKM/TPM were reserved for descriptive summaries and visualization.

Differentially expressed genes (DEGs) were identified with DESeq2 (v1.x). Genes with low counts (< 10 total across all samples) were filtered prior to modeling. Size factors were estimated by the median‐of‐ratios method, and dispersions were fit with the parametric trend. Wald tests were used for pairwise contrasts; p‐values were adjusted by Benjamini–Hochberg. Unless specified otherwise, significance was defined as |log_2_FC| ≥1 and FDR ≤ 0.05. In addition, KEGG pathway annotation was performed for the DEGs to summarize their functional categories relative to the whole‐transcriptome background. Annotations were derived from the Kyoto Encyclopedia of Genes and Genomes.

For visualization and QC, counts were transformed using a variance‐stabilizing transformation (VST) or rlog (DESeq2). PCA was computed on centered transformed values; score plots display 95% confidence ellipses per group. Sample‐to‐sample correlation (Pearson on VST/rlog) was shown as clustered heatmaps (Euclidean distance, complete linkage) with row/column dendrograms.

### 4.5 Gene Set Enrichment Analysis (GSEA)

For the bulk RNA‐seq comparison between NAD(H)‐treated and control groups, we used GSEA Desktop v4.3.2 (Broad Institute) in expression‐matrix mode. Genes were ranked by Signal2Noise. Gene sets were taken from MSigDB Mouse Collections v2025.1. Mm (MH Hallmark) [55]. Probe collapsing was disabled (No_collapse). The weighted enrichment statistic (p = 1) and 1,000 gene_set permutations were applied, with gene set size constrained to 15–500. Following standard GSEA guidance, we report FDR < 0.25 as the discovery threshold (appropriate for competitive GSEA with inter‐gene correlation and small‐n two‐class designs) and *p* < 0.05 as a secondary indicator [56].

### 4.6 Extraction of Rat Serum

8‐week‐old Sprague‐Dawley rats were obtained from the Animal Experiment Center of the Beijing Institute of Stem Cell and Regenerative Medicine. After isoflurane (RWD, R510‐22, China) sedation, anesthesia was induced via intraperitoneal injection of 5% chloral hydrate (Meilunbio, MB4202250G, China). Animals were positioned supine on a surgical platform, followed by abdominal disinfection with 75% ethanol. A midline laparotomy was performed to expose abdominal viscera, which were gently retracted using sterile gauze to visualize the abdominal aorta. The aorta was secured with vascular clamps, and a collection needle was inserted centripetally into the vessel. Whole blood samples were immediately aspirated into 10 mL vacuum tubes (BD, 367820, USA). Collected blood underwent coagulation for 30 min at room temperature prior to centrifugation (3000 × g, 4°C, 30 min). Serum supernatant was subsequently heat‐inactivated by incubation in distilled water at 56°C for 30 min.

### 4.7 E7.5 Embryo Dissection and IVC

E7.5 mouse embryos were harvested from pregnant mice euthanized via cervical dislocation without hormonal pretreatment. Embryos were incubated in DMEM (Gibco, 11330057, USA) supplemented with 10% fetal bovine serum (Invitrogen, 10437085, USA) at 37°C for 1 h. The solution was filtered through a 0.22 µm sterilizing membrane (Millipore, SLGPR33RB, USA) before use. Under microscopic guidance, using a Tokai Hit thermo plate (37°C), the uterine decidua was dissected and bisected along the embryonic long axis. Embryos were gently extracted, followed by the removal of the parietal yolk sac with fine forceps.

The IVC medium contained 50% CMRL 1066 medium (Gibco, 11530037, USA) and 50% rat serum, supplemented with 1% penicillin/streptomycin (Gibco, 15140122, USA). In the experimental groups, different small molecules at varying concentrations were added to the IVC medium. For static culture, embryos were placed in 4‐well cell culture plates (SPL Life Sciences, 30004–30, South Korea) containing IVC medium and maintained at 37°C under a humidified, low‐oxygen gas mixture (90% N_2_, 5% O_2_, 5% CO_2_). For roller culture, embryos were placed together with IVC medium in sterilized glass bottles mounted on a rotating drum inside a temperature‐controlled incubator at 37°C. The bottles were rotated continuously to keep the embryos in gentle suspension and to ensure uniform exposure to nutrients and gases. A continuous flow of humidified low‐oxygen gas (90% N_2_, 5% O_2_, 5% CO_2_) was supplied through the drum and evenly distributed into the culture bottles, maintaining stable oxygenation and pH throughout the culture period. For both static and roller culture, the medium was refreshed every 24 h by replacing 50% of the total volume with freshly prepared IVC medium to maintain nutrient and pH stability. All procedures followed institutional animal care guidelines, aiming to minimize animal distress. Approval was obtained from the relevant ethics committee responsible for this work.

### 4.8 Paraffin Sections and Hematoxylin‐Eosin (H&E) Staining

The IVC embryonic tissue samples were fixed in 4% paraformaldehyde for ≥24 h. The tissues were dehydrated sequentially in graded ethanol (50%, 70%, 80%, 95%, 100%, 100%) at room temperature (1 h per concentration). The tissues were cleared using a 1:1 mixture of 100% ethanol and xylene (30 min), followed by xylene until transparency was achieved.

The cleared tissues were infused with paraffin wax over three cycles (Wax I, II, III; 2 h each). They were embedded using an embedding machine (Leica, Germany) and sectioned into 5 µm slices with a microtome (Leica, Germany). The sections were deparaffinized in xylene (I, II, III; 10 min each) and rehydrated through descending ethanol gradients (100%, 95%, 85%, 75%; 5 min each).

Next, the sections were stained with hematoxylin (Bioroyee, RL3406, China; 4 min), rinsed in tap water (10 min), and differentiated in 1% HCl/70% ethanol (4 s). After rinsing again for 4 min, the sections were counterstained with a 0.5% eosin alcohol solution (Bioroyee, RL3417, China) for 8 min, then briefly rinsed for 10 s. The sections were then dehydrated through ascending ethanol (75%, 95%, 100%) and xylene (I, II, III; 5 min each). Finally, coverslips were mounted, and slides were imaged using a panoramic histocytometer (Leica, Germany) after drying.

### 4.9 mESCs Culture and Differentiation

The cultivation of mESCs and the subsequent differentiation in a system were performed using methods adapted from those established by Olivier Pourquie et al. [57]. Briefly, the mESCs were maintained under feeder‐free conditions in 0.1% gelatin (Stemcell Technologies, 07903, Canada)‐coated dishes with 2i medium composed of DMEM (Gibco, 11965118, USA) supplemented with 1% GlutaMAX (Gibco, 35050061, USA), 1% sodium pyruvate (Gibco, 11360070, USA), 1% non‐essential amino acids (Gibco, 11140050), 0.01% bovine serum albumin (BSA) (Sigma, A9205, USA), 0.1% β‐mercaptoethanol (Gibco, 21985023, USA), 15% fetal bovine serum (FBS) (Gibco, A5669701, USA), 1,000 U/mL leukemia inhibitory factor (LIF) (MedChemExpress, HY‐P78324c), 3 µm CHIR99021 (MedChemExpress, HY‐10182, China), and 1 µm PD0325901 (MedChemExpress, HY‐10254, China). Mouse ES cells were passaged by Accutase (Stemcell Technologies, 07903, Canada) dissociation every 2 days at a density of 1 × 10^4^ cells/cm^2^.

For the induction of PSM‐like progenitor cells, mESCs were seeded at a density of 1 × 10^4^ cells/cm^2^ in fibronectin‐coated dishes (BioCoat, 356008, USA) in N2B27 medium (composed of 50% neurobasal medium (Gibco, 21103049, USA), 50% DMEM/F‐12 (Gibco, 11320033, USA), 1% GlutaMAX (Gibco, 35050061, USA), 1% penicillin‐streptomycin (Gibco, 15140122, USA), 0.5% N‐2 supplement (Gibco, 17502048, USA), 1% B‐27 supplement (Gibco, 17504044, USA), 0.1% β‐mercaptoethanol (Gibco, 21985023, USA), and 0.01% BSA (Sigma, A9205, USA)) supplemented with 25 ng/mL activin A (MedChemExpress, HY‐P700159AF, China) and 12 ng/mL bFGF (MedChemExpress, HY‐P7066, China). After 48 h in culture, the differentiation medium was changed to DMEM (Gibco, 11965118, USA) supplemented with 1% GlutaMAX (Gibco, 35050061, USA), 1% sodium pyruvate (Gibco, 11360070, USA), 1% non‐essential amino acids (Gibco, 11140050, USA), 0.01% BSA (Sigma, A9205, USA), 0.1% β‐mercaptoethanol (Gibco, 21985023, USA), 15% FBS (Gibco, A5669701, USA), 3 µm CHIR99021 (MedChemExpress, HY‐10182, China), and 0.5 µm LDN193189 (Beyotime, SF7912, China). The cells were cultured for an additional 4 days, with daily medium changes.

### 4.10 Whole‐Mount Immunofluorescent Staining

Embryos were fixed in 4% paraformaldehyde for 1 h at room temperature, followed by three washes with phosphate‐buffered saline (PBS). Subsequently, embryos were dehydrated using a series of ethanol concentrations (25%, 50%, 75%, and 100%, twice) in the presence of 1% Triton X‐100 (Sigma, T8787) for 1.5–2 h at room temperature. Next, the embryos were blocked with 5% bovine serum albumin (BSA) for 2 h at room temperature. The primary antibody iFluor 488 phalloidin was incubated overnight at 4°C. After washing with PBS, the embryos were incubated overnight at 4°C with the DAPI stain solution (YEASEN, 40728ES03). The embryos were then washed with PBS and dehydrated through a methanol series (25%, 50%, 75%, and 100%, twice for 10 min each) before being immersed in a 1:2 benzyl alcohol: benzyl benzoate (BABB) solution. Finally, the embryos were mounted on 5 mm thick microscope slides (Easybio, 188105w) with BABB and covered with a coverslip. The images were acquired using a Zeiss LSM880 Fast Airyscan with Carl Zeiss ZEN 2011 software.

### 4.11 Immunofluorescence (IF) Staining

mESCs or embryonic tissue slices were washed three times for 5 min each and fixed with 4% paraformaldehyde for 30 min to 2 h. Cells were permeabilized with 0.5% Triton X‐100 for 30 min. Block with 3% BSA for 1 h. The primary antibodies, anti‐SOX2 (1:100, Cell Signaling Technology, 3579S, USA), anti‐TBX6 (1:100, R&D Systems, AF4744, USA), anti‐T (1:100, R&D Systems, AF2085, USA), anti‐CDX2 (1:100, Cell Signaling Technology, 12306S, USA), anti‐KI67 (1:100, Beyotime, C2312S, China), anti‐laminin (1:200, MilliporeSigma, L9393, USA), anti‐MCT1 (1:200, Proteintech, 20139‐1‐AP, USA) and anti‐PLF (1:200, Santa Cruz Biotechnology, sc‐47347, USA) were diluted in 5% BSA and incubated overnight at 4°C. Wash cells gently with PBS three times. Dilute secondary antibodies (1:200 in PBS) and incubate cells at room temperature for 1 h. Images were obtained using Zeiss LSM880 Fast Airyscan and LEICA confocal microscope (STELLARIS 5) and processed with ZEN and LAS X software.

### 4.12 Real‐Time Quantitative PCR (RT‐qPCR)

The total RNA was purified by TRIzol Reagent (Invitrogen, 15596018, USA). The RNA purity was confirmed by measuring the absorbance at 260/280 nm. The oligonucleotide primers of genes were constructed by TsingkeBiotechnologyCo., Ltd. (Beijing, China). 18S was used as an endogenous control. The cDNAs were synthesized using HiScript III All‐in‐one RT SuperMix Perfect for qPCR (Vazyme, R333‐01, China). The qPCR analyses for target genes were processed with the TB Green Premix Ex Taq II (Tli RNaseH Plus) (TaKaRa, RR820DS, Japan) on a LightCycler480 System (Roche Diagnostics, USA). The relative expression of each gene was calculated by 2^−△△Ct^ and each group had three technical replicates. Primer sequences used for RT‐qPCR of genes are shown in Table S5.

### 4.13 NAD^+^/NADH and Extracellular Lactate/Pyruvate Measurements

Whole‐cell NAD^+^ and NADH contents and their ratio in PSM‐like progenitor cells were quantified using the NAD^+^/NADH Assay Kit (Beyotime, S0175, China). For each biological replicate, approximately 1 × 10^6^ cells were harvested, the culture medium was removed, and cells were lysed in 200 µL of kit extraction buffer with thorough pipetting. Lysates were clarified at 12,000 × g, 4°C, 5–10 min, and supernatants were used for analysis. According to the manufacturer's instructions, an NADH standard curve (0–10 µm; recommended points: 0, 0.25, 0.5, 1, 2, 4, 6, 8, and 10 µm) was prepared in extraction buffer; 20 µL of standards or samples were dispensed into each well of a 96‐well plate. For total NAD (NAD_total), 90 µL ethanol dehydrogenase working solution (freshly prepared, enzyme: buffer 1:45) was added to each well and incubated at 37°C, protected from light, for 10 min, followed by 10 µL chromogenic solution (WST‐8/1‐mPMS). Plates were incubated at 37°C, protected from light, for 10–20 min and read at 450 nm. For NADH alone, 50–100 µL of the same sample was transferred to a 1.5 mL tube and heated at 60°C for 30 min to degrade NAD^+^, then processed identically for color development and reading. Concentrations were calculated as [NAD^+^] = [NAD_total] − [NADH] and [NAD^+^]/[NADH] = ([NAD_total] − [NADH]) / [NADH]. Extracellular lactate and pyruvate in conditioned media were measured using commercial ELISA kits (lactate: Signalway Antibody, EK18932, USA; pyruvate: Bioroyee, RE3955, China). Supernatants were collected 24 h after NADH treatment of PSM‐like progenitor cells, clarified at 1,200 × g, 4°C, 5 min to remove cells and debris, and diluted as recommended. Standard curves were prepared from the kit‐supplied lactate and pyruvate standards. Samples and standards were added to coated plates, incubated and washed according to the instructions, developed with substrate, and read at 450 nm.

### 4.14 Cell Counting Kit‐8 (CCK‐8) and Methylthiazolyldiphenyl‐tetrazolium Bromide (MTT) assays

Cell viability was assessed using the CCK‐8 (MedChemExpress, HY‐K0301, China) and MTT (Beyotime, C0009S, China) assays. For the CCK‐8 assay, cells were seeded in 96‐well plates at a density of 5000 cells per well and incubated with NADH for 24 h. After removing the NADH, 10 µL of CCK‐8 reagent was added to each well, and the cells were incubated for 4 h at 37°C in a humidified atmosphere containing 5% CO_2_. The absorbance was measured at 450 nm using a microplate reader (BioTek, Synergy2). For the MTT assay, cells were similarly seeded in 96‐well plates and treated as described above. Following treatment, 10 µL of 5 mg/mL MTT solution was added to each well and incubated for 4 h at 37°C. The supernatant was then carefully removed, and 100 µL of Formazan solution was added. The absorbance was measured at 570 nm using a microplate reader.

### 4.15 Statistical Analysis

Statistical analyses were performed using SPSS v20.0 (IBM Corp., Armonk, NY, USA) and GraphPad Prism v9.0 for general analyses. All tests were pre‐specified and two‐sided unless stated otherwise. Data are presented as mean ± SEM. Where applicable, data were inspected for outliers using pre‐defined criteria, and any transformation or normalization procedures are described in the corresponding Methods sections. Homogeneity of variance was assessed using Levene's test. For comparisons between two groups, unpaired two‐tailed Student's *t*‐tests were used, with Welch's correction applied when variances were unequal. For comparisons involving more than two groups, one‐way ANOVA followed by Tukey's post hoc multiple‐comparisons test was applied. Statistical significance was defined as a two‐sided *p* < 0.05. The exact sample size (n), experimental unit, and replicate structure for each experiment are provided in the corresponding figure legends. For high‐throughput omics analyses (metabolomics and transcriptomics), multiple testing was controlled using the FDR, with thresholds reported for each analysis.

## Author Contributions

G.C., Z.C.L., M.Y.W., Q.L., L.W., and K.Y.Y contributed equally to this work. J.L.Z., conceived and designed the project together with G.C., N. S.‐C., and H.M.W.; G.C., Z.C.L., M.Y.W., Q.L., L.W., and K.Y.Y contributed equally to experimental design, execution, and manuscript preparation. K.Y.Y., Q.L., L.W., D.N.Y., and W.W.M provided technical assistance and participated in data discussion. G.C., Z.C.L., and M.Y.W. wrote the first draft of the manuscript. N. S.‐C., J.L.Z., and H.M.W. supervised the study and revised the manuscript.

## Funding

This work was financially supported by the National Key Research and Development Program of China (2021YFA0805701, 2022YFA1104300, 2022YFA1103100), the National Natural Science Foundation of China (82322026, 82402032), the Postdoctoral Fellowship Program of CPSF (GZB20240738), the Beijing Nova Program (20240484730), the Strategic Priority Research Program of the Chinese Academy of Sciences (XDB0820000), the Beijing Natural Science Foundation (5254026) and the National Key Research and Development Program of China (2024YFA1803002, 2024YFA1802200).

## Conflicts of Interest

The authors declare no conflict of interest.

## Supporting information




**Supporting File 1**: advs74069‐sup‐0001‐SuppMat.docx.

**Supporting File 2**: advs74069‐sup‐0002‐TableS1.xlsx.

**Supporting File 3**: advs74069‐sup‐0003‐TableS2.xlsx.

**Supporting File 4**: advs74069‐sup‐0004‐TableS3.xlsx.

**Supporting File 5**: advs74069‐sup‐0005‐TableS4.xlsx.

**Supporting File 6**: advs74069‐sup‐0006‐TableS5.docx.

**Supporting File 7**: advs74069‐sup‐0007‐VideoS1.mp4.

**Supporting File 8**: advs74069‐sup‐0008‐VideoS2.mp4.

## Data Availability

The data that support the findings of this study are available in the supplementary material of this article.

## References

[1] S. A. Morris , Y. Guo , and M. Zernicka‐Goetz , “Developmental Plasticity Is Bound by Pluripotency and the Fgf and Wnt Signaling Pathways,” Cell Reports 2, no. 4 (2012): 756–765, 10.1016/j.celrep.2012.08.029. 23041313 PMC3607220

[2] S. Guo , X. Cui , X. Jiang , et al., “Tracing the Origin of the Placental Trophoblast Cells in Mouse Embryo Development†,” Biology of Reproduction 102, no. 3 (2020): 598–606, 10.1093/biolre/ioz201. 31621828

[3] K. Piotrowska‐Nitsche , A. Perea‐Gomez , S. Haraguchi , and M. Zernicka‐Goetz , “Four‐Cell Stage Mouse Blastomeres Have Different Developmental Properties,” Development (Cambridge, England) 132, no. 3 (2005): 479–490, 10.1242/dev.01602. 15634695

[4] D. Liu , Y. Chen , Y. Ren , et al., “Primary Specification of Blastocyst Trophectoderm by scRNA‐Seq: New Insights into Embryo Implantation,” Science Advances 8, no. 32 (2022): abj3725, 10.1126/sciadv.abj3725. PMC9365277 35947672

[5] B. Pijuan‐Sala , J. A. Griffiths , C. Guibentif , et al., “A Single‐Cell Molecular Map of Mouse Gastrulation and Early Organogenesis,” Nature 566, no. 7745 (2019): 490–495, 10.1038/s41586-019-0933-9. 30787436 PMC6522369

[6] X. Jiang , Y. Wang , Z. Xiao , et al., “A Differentiation Roadmap of Murine Placentation at Single‐cell Resolution,” Cell Discovery 9, no. 1 (2023): 30, 10.1038/s41421-022-00513-z. 36928215 PMC10020559

[7] L. Woods , V. Perez‐Garcia , and M. Hemberger , “Regulation of Placental Development and Its Impact on Fetal Growth‐New Insights from Mouse Models,” Frontiers in Endocrinology 9 (2018): 570, 10.3389/fendo.2018.00570. 30319550 PMC6170611

[8] S. A. Bainbridge , A. Minhas , K. J. Whiteley , et al., “Effects of Reduced Gcm1 Expression on Trophoblast Morphology, Fetoplacental Vascularity, and Pregnancy Outcomes in Mice,” Hypertension 59, no. 3 (2012): 732–739, 10.1161/HYPERTENSIONAHA.111.183939. 22275534

[9] S. Kaiser , Y. Koch , E. Kühnel , et al., “Reduced Gene Dosage of Tfap_2_c Impairs Trophoblast Lineage Differentiation and Alters Maternal Blood Spaces in the Mouse Placenta1,” Biology of Reproduction 93, no. 2 (2015): 1–13, 10.1095/biolreprod.114.126474. 26063869

[10] S. A. Elmore , R. Z. Cochran , B. Bolon , et al., “Histology Atlas of the Developing Mouse Placenta,” Toxicologic Pathology 50, no. 1 (2022): 60–117, 10.1177/01926233211042270. 34872401 PMC8678285

[11] B. Marsh and R. Blelloch , “Single Nuclei RNA‐seq of Mouse Placental Labyrinth Development,” Elife 9 (2020): 60266, 10.7554/eLife.60266. PMC7669270 33141023

[12] E. D. Watson and J. C. Cross , “Development of Structures and Transport Functions in the Mouse Placenta,” Physiology (Bethesda, Md) 20 (2005): 180–193, 10.1152/physiol.00001.2005. 15888575

[13] S. Panja and B. C. Paria , “Development of the Mouse Placenta,” Advances in Anatomy, Embryology and Cell Biology 234 (2021): 205–221, 10.1007/978-3-030-77360-1_10. 34694483 PMC9109784

[14] E. D. Watson and J. C. Cross , “Development of Structures and Transport Functions in the Mouse Placenta,” Physiology 20, no. 3 (2005): 180–193, 10.1152/physiol.00001.2005. 15888575

[15] I. L. M. H. Aye , S. Gong , G. Avellino , et al., “Placental Sex‐dependent Spermine Synthesis Regulates Trophoblast Gene Expression through Acetyl‐coA Metabolism and Histone Acetylation,” Communications Biology 5, no. 1 (2022): 586, 10.1038/s42003-022-03530-6. 35705689 PMC9200719

[16] A. Bonnin , N. Goeden , K. Chen , et al., “A Transient Placental Source of Serotonin for the Fetal Forebrain,” Nature 472, no. 7343 (2011): 347–350, 10.1038/nature09972. 21512572 PMC3084180

[17] M. J. Jeyarajah , V. S. Patterson , G. Jaju Bhattad , L. Zhao , S. N. Whitehead , and S. J. Renaud , “Placental Extracellular Vesicles Promote Cardiomyocyte Maturation and Fetal Heart Development,” Communications Biology 7, no. 1 (2024): 1254, 10.1038/s42003-024-06938-4. 39363116 PMC11450004

[18] E. M. George , J. P. Warrington , F. T. Spradley , A. C. Palei , and J. P. Granger , “The Heme Oxygenases: Important Regulators of Pregnancy and Preeclampsia,” American Journal of Physiology‐Regulatory, Integrative and Comparative Physiology 307, no. 7 (2014): R769–R777, 10.1152/ajpregu.00132.2014. 24898840 PMC4187186

[19] M. L. Zenclussen , N. Linzke , A. Schumacher , et al., “Heme Oxygenase‐1 Is Critically Involved in Placentation, Spiral Artery Remodeling, and Blood Pressure Regulation during Murine Pregnancy,” Frontiers in pharmacology 5 (2014): 291, 10.3389/fphar.2014.00291. 25628565 PMC4292788

[20] R. Karahoda , C. Abad , H. Horackova , et al., “Dynamics of Tryptophan Metabolic Pathways in Human Placenta and Placental‐Derived Cells: Effect of Gestation Age and Trophoblast Differentiation,” Frontiers in Cell and Developmental Biology 8 (2020): 574034, 10.3389/fcell.2020.574034. 33072756 PMC7530341

[21] A. Bednov , J. Espinoza , A. Betancourt , Y. Vedernikov , M. Belfort , and C. Yallampalli , “L‐Arginine Prevents Hypoxia‐induced Vasoconstriction in Dual‐Perfused human Placental Cotyledons,” Placenta 36, no. 11 (2015): 1254–1259, 10.1016/j.placenta.2015.08.012. 26342955

[22] A. Solmonson , B. Faubert , W. Gu , et al., “Compartmentalized Metabolism Supports Midgestation Mammalian Development,” Nature 604, no. 7905 (2022): 349–353, 10.1038/s41586-022-04557-9. 35388219 PMC9007737

[23] Y. Xu , W. Xie , and J. Zhang , “Metabolic Regulation of Key Developmental Events during Mammalian Embryogenesis,” Nature Cell Biology 27, no. 8 (2025): 1219–1229, 10.1038/s41556-025-01720-y. 40696105

[24] S. Bruzzone , L. Guida , E. Zocchi , L. Franco , and A. D. Flora , “Connexin 43 Hemichannels Mediate Ca2+‐Regulated Transmembrane NAD+ Fluxes in Intact Cells,” The FASEB Journal 15, no. 1 (2001): 10–12, 10.1096/fj.00-0566fje. 11099492

[25] R. A. Billington , C. Travelli , E. Ercolano , et al., “Characterization of NAD Uptake in Mammalian Cells,” Journal of Biological Chemistry 283, no. 10 (2008): 6367–6374, 10.1074/jbc.M706204200. 18180302

[26] M. Ziegler , M. Monné , A. Nikiforov , G. Agrimi , I. Heiland , and F. Palmieri , “Welcome to the Family: Identification of the NAD+ Transporter of Animal Mitochondria as Member of the Solute Carrier Family SLC25,” Biomolecules 11, no. 6 (2021): 880, 10.3390/biom11060880. 34198503 PMC8231866

[27] L. Liu , X. Su , W. J. Quinn , et al., “Quantitative Analysis of NAD Synthesis‐Breakdown Fluxes,” Cell Metabolism 27, no. 5 (2018): 1067–1080, 10.1016/j.cmet.2018.03.018. 29685734 PMC5932087

[28] F. J. Rosario , K. Barentsen , T. L. Powell , et al., “Trophoblast‐specific Overexpression of the LAT1 Increases Transplacental Transport of Essential Amino Acids and Fetal Growth in Mice,” PNAS Nexus 3, no. 6 (2024): pgae207, 10.1093/pnasnexus/pgae207. 38894879 PMC11184900

[29] P. R. Chen , C. G. Lucas , R. F. Cecil , et al., “Disrupting Porcine Glutaminase Does Not Block Preimplantation Development and Elongation nor Decrease mTORC1 Activation in Conceptuses,” Biology of Reproduction 105, no. 5 (2021): 1104–1113, 10.1093/biolre/ioab165. 34453429

[30] R. Lujan , R. Shigemoto , and G. Lopez‐Bendito , “Glutamate and GABA Receptor Signalling in the Developing Brain,” Neuroscience 130, no. 3 (2005): 567–580, 10.1016/j.neuroscience.2004.09.042. 15590141

[31] A. Špirková , V. Kovaříková , Z. Šefčíková , et al., “Glutamate Can Act as a Signaling Molecule in Mouse Preimplantation Embryos,” Biology of Reproduction 107 (2022): 916–927, 10.1093/biolre/ioac126. 35746896 PMC9562114

[32] P. R. Chen , B. K. Redel , L. D. Spate , T. Ji , S. R. Salazar , and R. S. Prather , “Glutamine Supplementation Enhances Development of in Vitro‐produced Porcine Embryos and Increases Leucine Consumption from the Medium†,” Biology of Reproduction 99, no. 5 (2018): 938–948, 10.1093/biolre/ioy129. 29860318 PMC6297286

[33] Y. I. Yaman and S. Ramanathan , “Controlling human Organoid Symmetry Breaking Reveals Signaling Gradients Drive Segmentation Clock Waves,” Cell 186, no. 3 (2023): 513–527, 10.1016/j.cell.2022.12.042. 36657441 PMC10025047

[34] M. F. Simsek , A. S. Chandel , D. Saparov , O. Q. H. Zinani , N. Clason , and E. M. Özbudak , “Periodic Inhibition of Erk Activity Drives Sequential Somite Segmentation,” Nature 613, no. 7942 (2023): 153–159, 10.1038/s41586-022-05527-x. 36517597 PMC9846577

[35] M. Diaz‐Cuadros , T. P. Miettinen , O. S. Skinner , et al., “Metabolic Regulation of Species‐specific Developmental Rates,” Nature 613, no. 7944 (2023): 550–557, 10.1038/s41586-022-05574-4. 36599986 PMC9944513

[36] D. H. Williamson , P. Lund , and H. A. Krebs , “The Redox state of Free Nicotinamide‐adenine Dinucleotide in the Cytoplasm and Mitochondria of Rat Liver,” Biochemical Journal 103, no. 2 (1967): 514–527, 10.1042/bj1030514. 4291787 PMC1270436

[37] M. Matsuda , J. Lázaro , and M. Ebisuya , “Metabolic Activities Are Selective Modulators for Individual Segmentation Clock Processes,” Nature Communications 16, no. 1 (2025): 845, 10.1038/s41467-025-56120-5. PMC11746943 39833174

[38] M. Diaz‐Cuadros and O. Pourquie , “The Clockwork Embryo: Mechanisms Regulating Developmental Rate,” Annual Review of Genetics 57 (2023): 117–134, 10.1146/annurev-genet-022123-104503. 38012023

[39] Y. Miao and O. Pourquié , “Cellular and Molecular Control of Vertebrate Somitogenesis,” Nature Reviews Molecular Cell Biology 25, no. 7 (2024): 517–533, 10.1038/s41580-024-00709-z. 38418851 PMC11694818

[40] B. K. Stepien , V. Pawolski , M. Wagner , T. Kurth , M. H. H. Schmidt , and H. Epperlein , “The Role of Posterior Neural Plate‐Derived Presomitic Mesoderm (PSM) in Trunk and Tail Muscle Formation and Axis Elongation,” Cells 12, no. 9 (2023): 1313, 10.3390/cells12091313. 37174713 PMC10177618

[41] M. Diaz‐Cuadros , T. P. Miettinen , O. S. Skinner , et al., “Metabolic Regulation of Species‐Specific Developmental Rates,” Nature 613, no. 7944 (2023): 550–557, 10.1038/s41586-022-05574-4. 36599986 PMC9944513

[42] C. Kappen , C. Kruger , S. Jones , and J. M. Salbaum , “Nutrient Transporter Gene Expression in the Early Conceptus—Implications from Two Mouse Models of Diabetic Pregnancy,” Frontiers in Cell and Developmental Biology 10 (2022): 777844, 10.3389/fcell.2022.777844. 35478964 PMC9035823

[43] S. Tohyama , F. Hattori , M. Sano , et al., “Distinct Metabolic Flow Enables Large‐Scale Purification of Mouse and human Pluripotent Stem Cell‐derived Cardiomyocytes,” Cell Stem Cell 12, no. 1 (2013): 127–137, 10.1016/j.stem.2012.09.013. 23168164

[44] T. C. Umei , S. Tohyama , and K. Fukuda , “Metabolism‐Based Cardiomyocytes Production for Regenerative Therapy,” Journal of Molecular and Cellular Cardiology 176, no. 1 (2023): 11–20, 10.1016/j.yjmcc.2023.01.007. 36681267

[45] M. Parenti , S. Rosen , M. Pettes , et al., “Multi‐omic Integration Reveals Dynamic Changes in Human Placental Metabolism Across Gestation,” bioRxiv (2025), 10.1101/2025.10.22.683996.

[46] R. Aouache , L. Biquard , D. Vaiman , and F. Miralles , “Oxidative Stress in Preeclampsia and Placental Diseases,” International Journal of Molecular Sciences 19, no. 5 (2018): 1496, 10.3390/ijms19051496. 29772777 PMC5983711

[47] S. Muralimanoharan , A. Maloyan , J. Mele , C. Guo , L. G. Myatt , and L. Myatt , “MIR‐210 Modulates Mitochondrial Respiration in Placenta with Preeclampsia,” Placenta 33, no. 10 (2012): 816–823, 10.1016/j.placenta.2012.07.002. 22840297 PMC3439551

[48] S. Mukherjee , J. L. James , B. Thilaganathan , G. S. Whitley , A. E. Michael , and J. E. Cartwright , “Elevated Glucocorticoid Metabolism in Placental Tissue from First Trimester Pregnancies at Increased Risk of Pre‐Eclampsia,” Placenta 32, no. 9 (2011): 687–693, 10.1016/j.placenta.2011.06.014. 21767875

[49] F. Jahan , G. Vasam , Y. Cariaco , et al., “NAD + Depletion Is central to Placental Dysfunction in an Inflammatory Subclass of Preeclampsia,” Life Science Alliance 7, no. 12 (2024): 202302505, 10.26508/lsa.202302505. PMC11467044 39389781

[50] X. Cheng , S. J. Chapple , B. Patel , et al., “Gestational Diabetes Mellitus Impairs Nrf_2_‐mediated Adaptive Antioxidant Defenses and Redox Signaling in Fetal Endothelial Cells in Utero,” Diabetes 62, no. 12 (2013): 4088–4097, 10.2337/db13-0169. 23974919 PMC3837032

[51] Z. Wang , T. Zhou , M. Shao , et al., “Maternal β‐Nicotinamide Mononucleotide Supplementation Reduces the IUGR Rate by Improving Mitochondrial Function in the Placenta of Sows via AMPK/PGC‐1α Pathway,” Molecular Nutrition & Food Research 69, no. 14 (2025): 70061, 10.1002/mnfr.70061. 40420689

[52] A. S. Pereyra , C. Lin , D. M. Sanchez , et al., “Skeletal Muscle Undergoes fiber Type Metabolic Switch without Myosin Heavy Chain Switch in Response to Defective Fatty Acid Oxidation,” Molecular Metabolism 59 (2022): 101456, 10.1016/j.molmet.2022.101456. 35150906 PMC8898976

[53] L. Wang , D. Li , F. Yao , et al., “Serpina3k Lactylation Protects from Cardiac Ischemia Reperfusion Injury,” Nature Communications 16, no. 1 (2025): 1012, 10.1038/s41467-024-55589-w. PMC11760901 39856050

[54] N. Shyh‐Chang , J. W. Locasale , C. A. Lyssiotis , et al., “Influence of Threonine Metabolism on S‐Adenosylmethionine and Histone Methylation,” Science 339, no. 6116 (2013): 222–226, 10.1126/science.1226603. 23118012 PMC3652341

[55] A. S. Castanza , J. M. Recla , D. Eby , H. Thorvaldsdóttir , C. J. Bult , and J. P. Mesirov , “Extending Support for Mouse Data in the Molecular Signatures Database (MSigDB),” Nature Methods 20, no. 11 (2023): 1619–1620, 10.1038/s41592-023-02014-7. 37704782 PMC11397807

[56] A. Subramanian , P. Tamayo , V. K. Mootha , et al., “Gene Set Enrichment Analysis: A Knowledge‐Based Approach for Interpreting Genome‐Wide Expression Profiles,” Proceedings of the National Academy of Sciences 102, no. 43 (2005): 15545–15550, 10.1073/pnas.0506580102. PMC1239896 16199517

[57] M. Diaz‐Cuadros , D. E. Wagner , C. Budjan , et al., “In Vitro Characterization of the human Segmentation Clock,” Nature 580, no. 7801 (2020): 113–118, 10.1038/s41586-019-1885-9. 31915384 PMC7336868

