# Supplementary material for: Dynamics of the Mammalian Placental Metabolome in Placentogenesis and Embryonic Development
Source: Adv Sci (Weinh). 2026 Jan 28;13(19):e07928. doi: 10.1002/advs.202507928 (PMC13045311; doi:10.1002/advs.202507928)
Supplement: Supplementary file 1 — Supporting File 1: advs74069‐sup‐0001‐SuppMat.docx. [file ADVS-13-e07928-s007.docx]

**Supporting Information**

**Title: Dynamics of Mammalian Placenta Metabolites in Placentogenesis and Embryonic Development**

*Gang Chen, Zichen Liu, Mengyi Wei, Qian Li, Liang Wu, Kunyuan Yu, Yanhong Xu, Dainan Yu, Wenwu Ma, Hongmei Wang,* *Ng Shyh-Chang* and Jinglei Zhai**


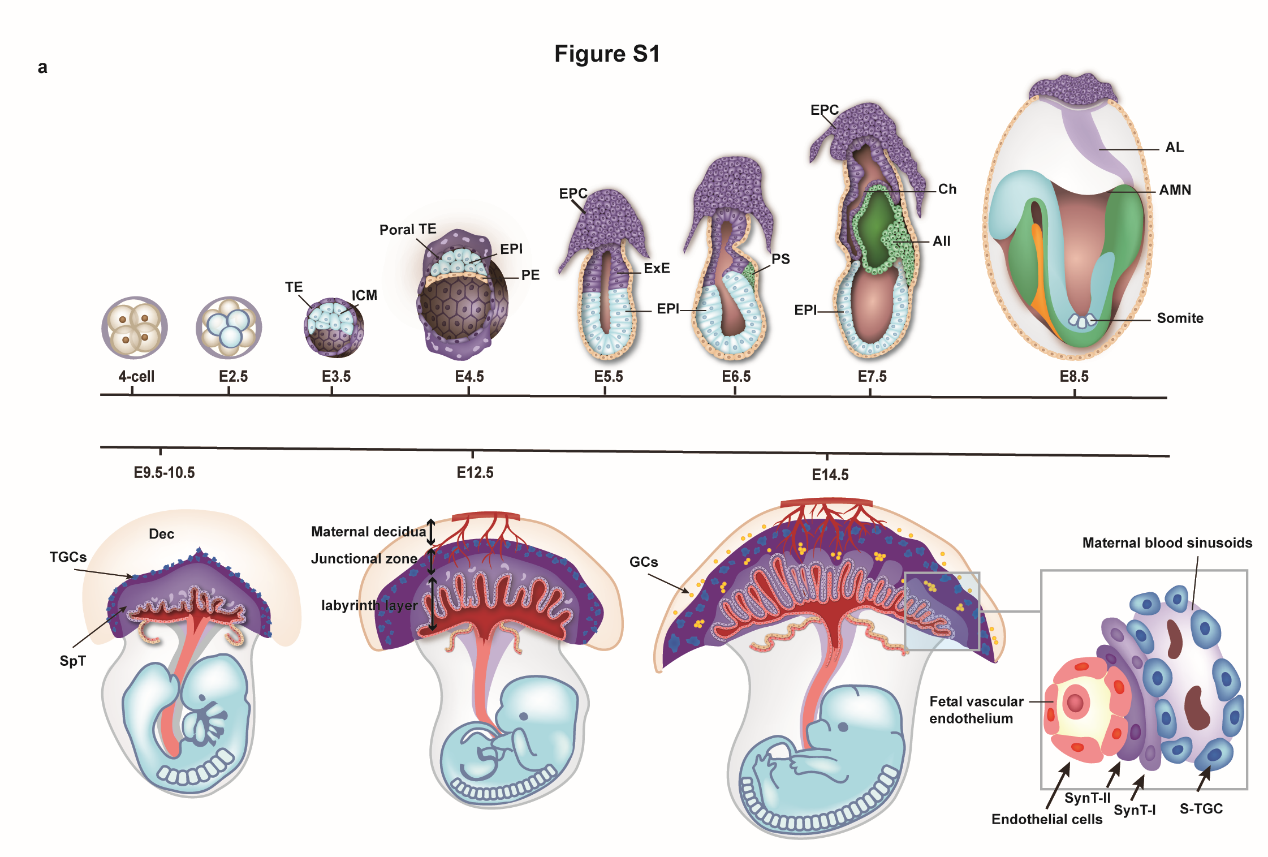


Figure S1: Schematic Overview of Mouse Embryonic and Placental Development from Preimplantation to Mid-gestation. The upper panel illustrates the key morphological transitions of the mouse embryo from the 4-cell stage through gastrulation (E6.5–E7.5) to early organogenesis (E8.5). The lower panel depicts progressive placental morphogenesis from E9.5 to E14.5, showing differentiation of trophoblast subtypes and the formation of maternal–fetal exchange structures. Abbreviations: ICM, inner cell mass. TE, trophectoderm. EPI, epiblast. PE, primitive endoderm. EXE, extraembryonic ectoderm. EPC, ectoplacental cone. PS, primitive streak. Ch, chorionic plate. AL, allantois. AMN, amnion. SpT, spongiotrophoblast. TGCs, Trophoblast Giant Cells. Dec, decidua. GCs, glycogen cells. SynT, syncytiotrophoblast. S-TGC, sinusoid-TGC.


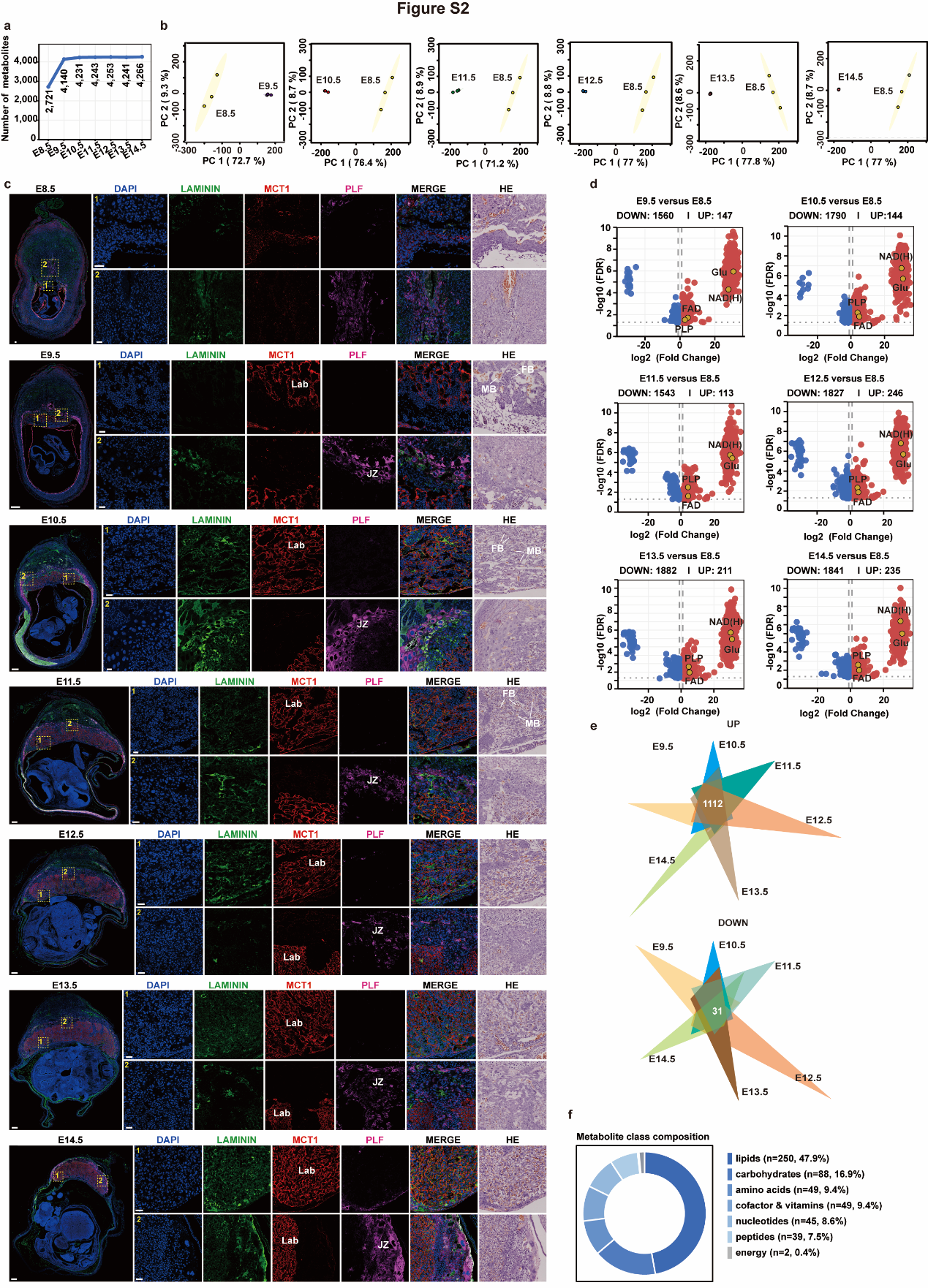


**Figure S2: Stage-resolved placental metabolomics and histology.** (a) Number of detected metabolites across E8.5–14.5 placentogenesis. (b) PCA results of metabolomic data at different developmental stages of the mouse placenta. Points represent samples. Per E8.5 sample contains 27-30 placentas from three litters, and per sample of E9.5-14.5 stage contains 9 placentas collected from three litters (please see details in Table S1). (c) Placental histology and immunofluorescence across stages (E8.5-14.5). Representative sections show nuclei (DAPI), monocarboxylate transporter-1 (MCT1, syncytiotrophoblast layer I marker), laminin (basement membrane marker), and PLF (proliferin; prolactin-family trophoblast marker), alongside hematoxylin–eosin (HE) staining. Lab: labyrinthine layer, JZ: junctional zone. FB: fetal blood, MB: maternal blood. Panels illustrate maturation of the labyrinthine exchange interface and the spongiotrophoblast compartment over time. Scale bars, 500 µm (left panels) and 50 µm (zoomed panels). (d) Differential metabolite landscapes relative to E8.5. Volcano plots for pairwise comparisons (E9.5 versus E8.5; E10.5 versus E8.5; E11.5 versus E8.5; E12.5 versus E8.5; E13.5 versus E8.5; E14.5 versus E8.5). The x-axis shows log_2_ (fold change) and the y-axis shows −log₁₀ (FDR). Counts of up- and down-regulated metabolites are showed in each panel. The horizontal reference corresponds to FDR = 0.05. (e) Stage-wise counts of up- and down-regulated metabolites for each contrast versus E8.5 (star plots). (f) Donut chart of global metabolite class composition by KEGG annotation (counts and percentages shown).


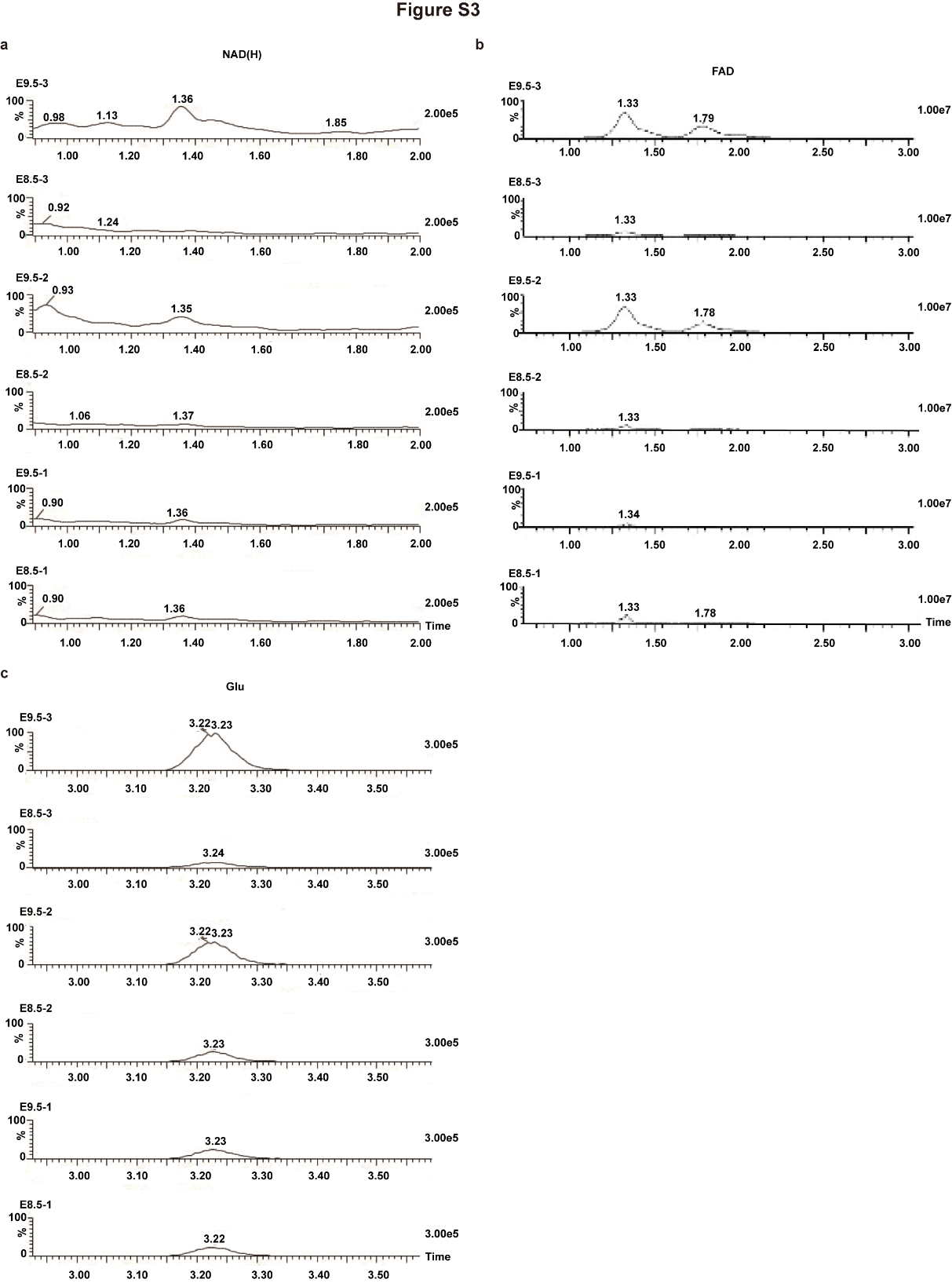


**Figure S3: LC-MS Total Ion Chromatograms of the Placenta Samples.** (a-c) Total ion chromatograms for (a) NAD(H), (b) FAD, and (c) L-glutamate (Glu) detected in placental extracts from E8.5 and E9.5 placentas. Each stage shows three technical replicates demonstrating reproducible retention times and peak shapes. The x-axis indicates retention time (min), and the y-axis indicates relative ion intensity.


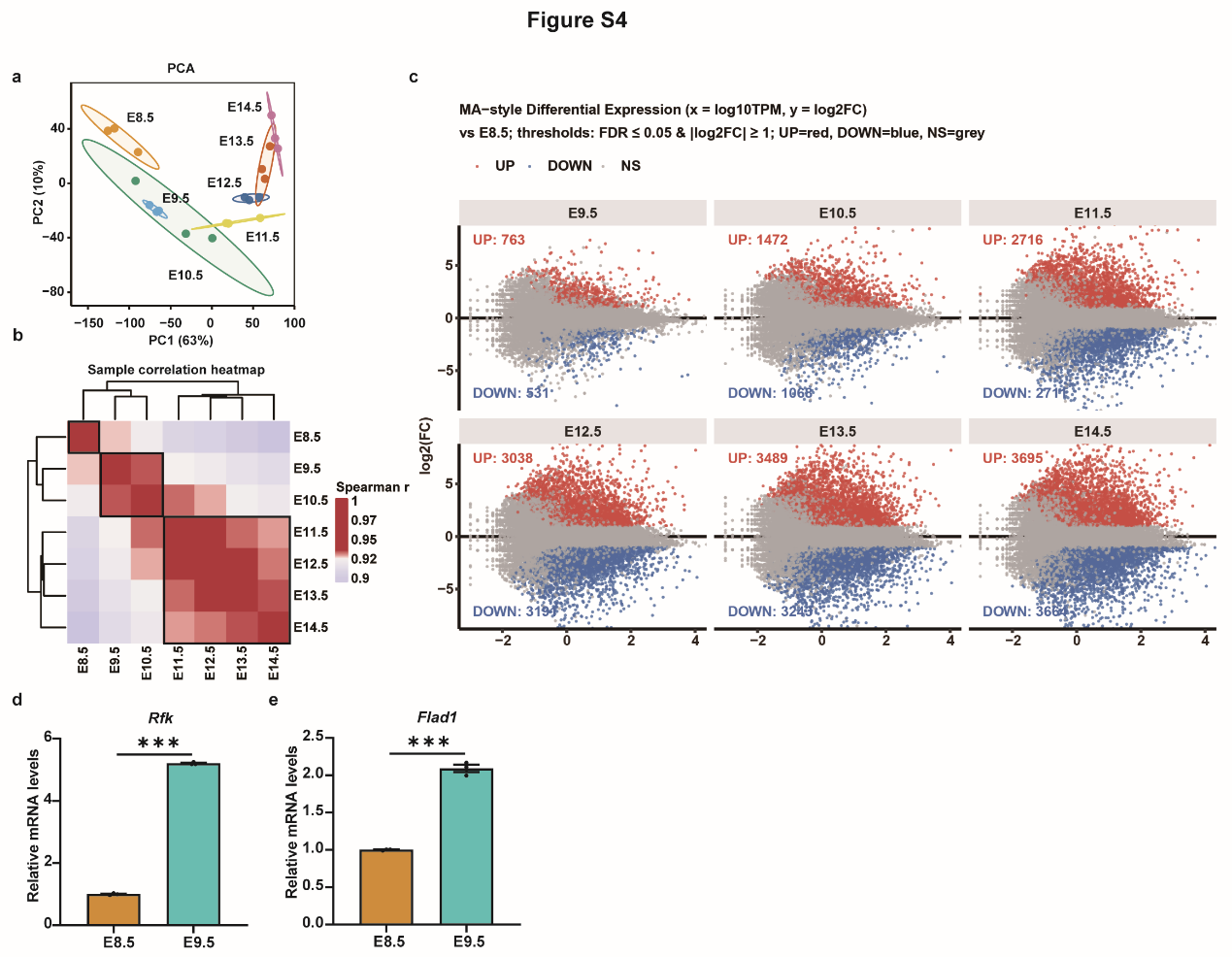


**Figure S4: Transcriptomics Analysis of Mouse E8.5-14.5 Placentogenesis.** (a) PCA results of transcriptomic data at different developmental stages of the mouse placenta. Points represent samples. Per E8.5 sample contains 29-31 placentas from three litters, and per sample of E9.5-14.5 stage contains 9 placentas collected from three litters (please see details in Table S1). (b) Spearman correlation heatmap of per-stage mean transcriptomes, showing sample-to-sample similarity across developmental stages. (c) MA-style differential expression plots comparing each stage with E8.5 (x: log10 TPM; y: log2FC; FDR ≤ 0.05 and |log2FC| ≥ 1). Red: upregulated. blue: downregulated. Gray, not significant. Gene counts listed on each panel. (d-e) qRT-PCR validation of riboflavin/FAD pathway genes *Rfk* (d) and *Flad1* (e), E9.5 versus E8.5 (mean ± s.e.m.; *P* < 0.001, two-tailed t-test).


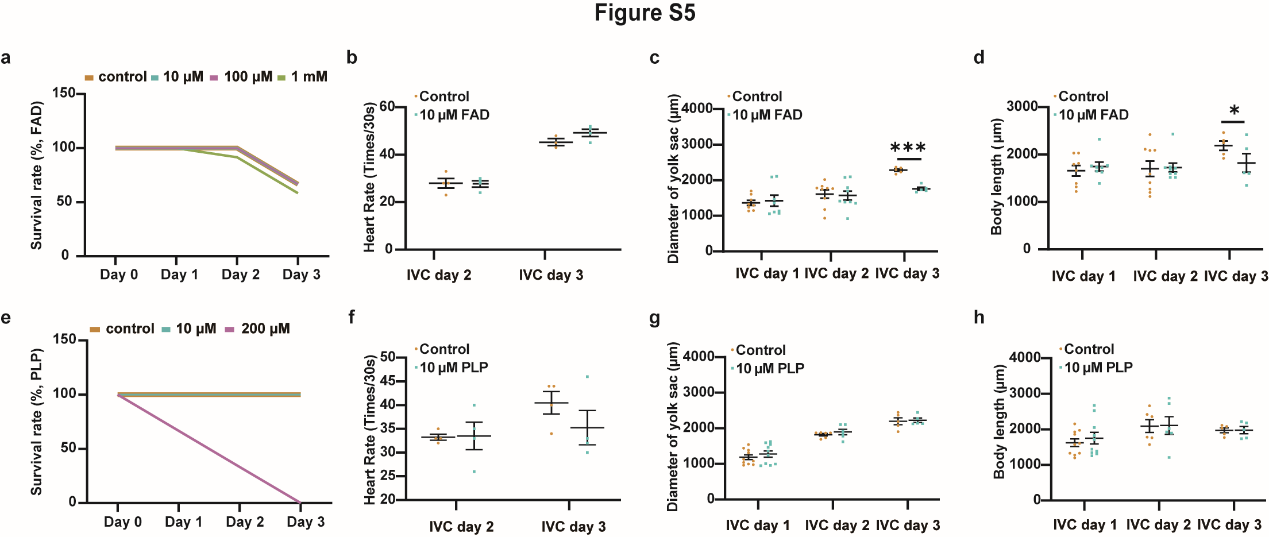


**Figure S5: The Effects of FAD and PLP on Embryonic Development.** (a) Survival curves of IVC embryos after FAD administration. n = 8. (b-d) Effects of FAD on (b) the heart rate, (c) the diameter of the yolk sac and (d) the body length of IVC embryos. Data are mean ± s.e.m. n = 8. Statistics: two-tailed unpaired Student’s t-test, *: *P*≤ 0.05. ***: *P* ≤ 0.001. (e) Survival curves of IVC embryos after PLP administration. n = 9. (f-h) Effects of PLP on (f) the heart rate, (g) the diameter of the yolk sac and (g) the body length of IVC embryos. Data are mean ± s.e.m. n = 9.


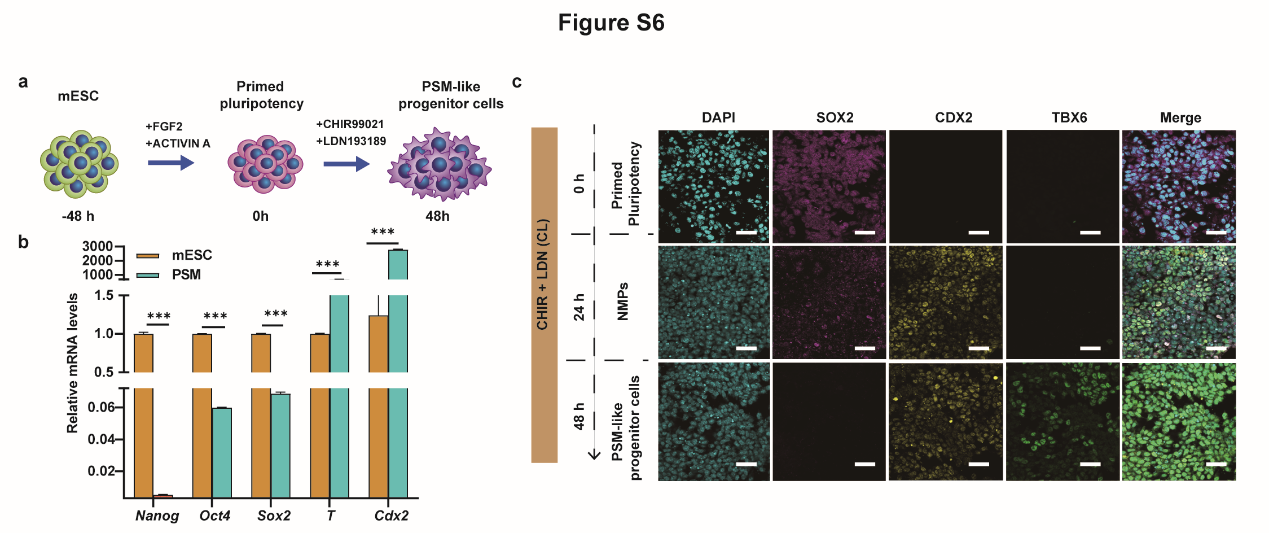


**Figure S6: The Induction of PSM-like progenitor cells from mESCs.** (a) Schematic representation of the stepwise induction of PSM-like progenitor cells from mESCs via primed pluripotency intermediates. (b) qRT–PCR analysis of pluripotency and mesodermal marker expression during PSM induction. Pluripotency genes (*Nanog*, *Oct4, Sox2*) were downregulated, whereas PSM-like cell markers (*T, Cdx2*) were significantly upregulated. Data are mean ± s.e.m. n = 3, Statistics: two-tailed unpaired Student’s t-test, ***: *P* ≤ 0.001. (c) Immunofluorescence analysis showing dynamic expression of SOX2, CDX2, and TBX6 during differentiation from mESCs to PSM-like progenitor cells (0 h: pluripotent state; 24 h: NMPs; 48 h: PSM-like progenitor cells. Scale bar = 50 μm.


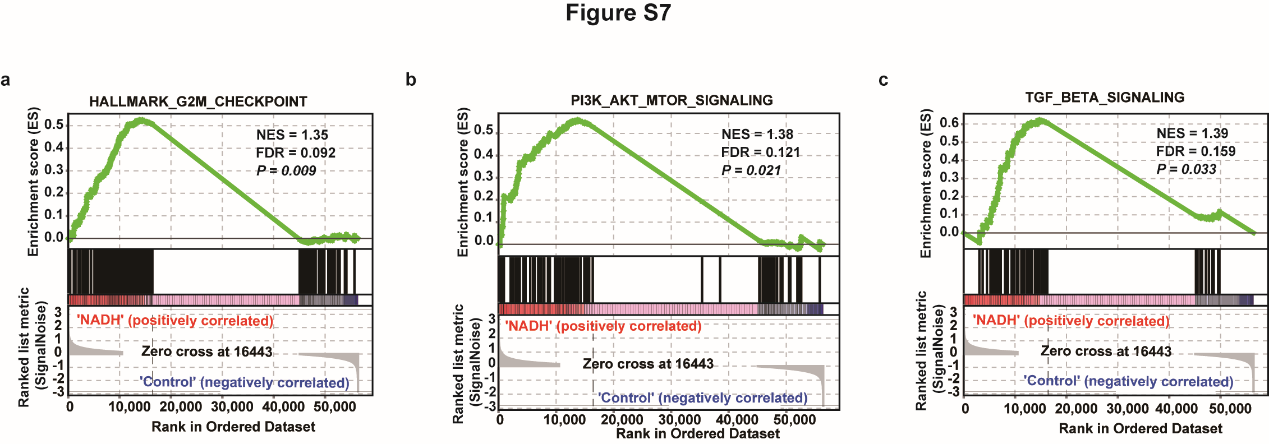


**Figure S7:** **GSEA identifies activation of proliferation-related pathways in NAD(H)-treated PSM-like progenitor cells.** Pre-ranked gene set enrichment analysis (GSEA) of RNA-seq data comparing NAD(H)-treated and control PSM-like progenitor cells. Enrichment plots for (a) HALLMARK_G2M_CHECKPOINT, (b) PI3K_AKT_MTOR_SIGNALING, and (c) TGF_BETA_SIGNALING are shown. Normalized enrichment scores (NES), false discovery rates (FDR), and nominal *P* values are indicated in each panel; the leading-edge subsets are shown below each curve.

**Tables:**

Table S1: Metabolome and Transcriptome Sequencing Sample Information, Including Gestation Stage, Sample Name and No. of Placentas Collected.

Table S2: Identification and Quantification of Metabolites in E8.5-14.5 Mouse Placentas, with Standardized Processing of Differential Metabolite Expression.

Table S3: The Analysis of Key Metabolites and Their Metabolic Pathways.

Table S4: Count Table for Mouse Placental Bulk RNA-seq (E8.5–E14.5).

Table S5: The Primers Sequences of RT-qPCR.

**Videos:**

**Video 1:** The Embryos Cultured *In Vitro*.

**Video 2:** The Whole-mount Immunofluorescent Staining of E8.5 Embryo.
