# Supplementary material for: Dynamics of the Mammalian Placental Metabolome in Placentogenesis and Embryonic Development
Source: Adv Sci (Weinh). 2026 Jan 28;13(19):e07928. doi: 10.1002/advs.202507928 (PMC13045311; doi:10.1002/advs.202507928)
Supplement: Supplementary file 6 — Supporting File 6: advs74069‐sup‐0006‐TableS5.docx. [file ADVS-13-e07928-s001.docx]

**Primers sequences**

| Primer | Sequence |
| --- | --- |
| *Ki67_F* | *ACCATCATTGACCGCTCCTTT* |
| *Ki67_R* | *AGGCCCTTGGCATACACAAA* |
| *Pcna_F* | *AAAGATGCCGTCGGGTGAAT* |
| *Pcna_R* | *TGGTTACCGCCTCCTCTTCT* |
| *18S_F* | *GGGACAAGGATAGTCATTTTGGGG* |
| *18S_R* | *TGTCATTGAGAGCAATGCCAGCCC* |
| *Ogdh_F* | *GTTTCTTCAAACGTGGGGTTCT* |
| *Ogdh_R* | *GCATGATTCCAGGGGTCTCAAA* |
| *Me2_F* | *GGCTAAGAGCTGTTACCACTCC* |
| *Me2_R* | *CGTAAACGCCATTCCCTTGTT* |
| *Aldh3a2-F* | *GATCTTGGCTGAACTCCTCCCT* |
| *Aldh3a2-R* | *GAGAATGTGGTCAAACCGCTGC* |
| *Aldh6a1-F* | *GCTCCTTGCTAAGTTGCTTCAGG* |
| *Aldh6a1-R* | *TACTCTCCTGCCTGGTTGGATC* |
| *Aldh7a1-F* | *CCTCGTTAGTGTGGCTGTCACA* |
| *Aldh7a1-R* | *AAGGACAGCAGGTTCACACGCT* |
| *Nanog_F* | *AGAAGTACCTCAGCCTCCAGC* |
| *Nanog_R* | *AGATGCGTTCACCAGATAGCC* |
| *Oct4_F* | *GAGGAAGCCGACAACAATGAG* |
| *Oct4_R* | *TGTGAGTGATCTGCTGTAGGGAG* |
| *Sox2_F* | *GGTTACCTCTTCCTCCCACTCCAGG* |
| *Sox2_R* | *TGTGCCGTTAATGGCCGTGCC* |
| *T_F* | *ACCTATGCGGACAATTCATC* |
| *T_R* | *CAGACCAGAGACTGGGATAC* |
| *Gapdh_F* | *GTGGCAAAGTGGAGATTGTTG* |
| *Gapdh_R* | *CTCCTGGAAGATGGTGATGG* |
| *Cdx2_F* | *AGGCTGAGCCATGAGGAGTA* |
| *Cdx2_R* | *TGTCTTCCCCTGAGGTCCAT* |
| *Rfk_F* | *CCCCACAGCCAATTTTCCTGA* |
| *Rfk_R* | *TGCTCACCACCATTTTATGGAC* |
| *Flad_F* | *AGGGACACACGCAAGATACC* |
| *Flad_R* | *GGTGGCTACTTCATCAGGCA* |
| *Bckdha-F* | *CTCCTGTTGGGACGATCTGG* |
| *Bckdha-R* | *CATTGGGCTGGATGAACTCAA* |
| *Idh3a-F* | *TGGGTGTCCAAGGTCTCTC* |
| *ldh3a-R* | *CTCCCACTGAATAGGTGCTTTG* |
| *Xdh-F* | *ATGACGAGGACAACGGTAGAT* |
| *Xdh-R* | *TCATACTTGGAGATCATCACGGT* |
| *Pdha2-F* | *CTGTCTCACGTATTTTCGGGAA* |
| *Pdha2-R* | *AGCCGGTACAGGTCACATTTC* |
| *Pdhb-F* | *AGGAGGGAATTGAATGTGAGGT* |
| *Pdhb-R* | *ACTGGCTTCTATGGCTTCGAT* |
| *Hadha-F* | *GTTTGAGGACCTCGGTGTAAAGC* |
| *Hadha-R* | *GAGAGCAGATGTGTTGCTGGCA* |
| *Hpgd-F* | *AAGCAAAACGGAGGTGAAGGCG* |
| *Hpgd-R* | *GAGCGTGTGAATCCGATGATGC* |
| *Impdh1-F* | *CTGTGGTTCCATCTGCATCACC* |
| *Impdh1-R* | *GATGCCACCATCCGCTATTACC* |
| *Ndufs7-F* | *GGCTGAGTATGTGGTGACCAAG* |
| *Ndufs7-R* | *AGCCATGTGCATCATCTCCACG* |
| *Glud1-F* | *TCCGTTACAGCACTGACGTGAG* |
| *Glud1-R* | *ACGCCTGCTTTAGCACCTCCAA* |
| *Aldh1a2-F* | *ATGGGTGAGTTTGGCTTACG* |
| *Aldh1a2-R* | *GGTTCATTGGAAGGCAGAAA* |
| *Ldha-F* | *TGTGGCAGACTTGGCTGAGA* |
| *Ldha-R* | *CTGAGGAAGACATCCTCATTGATTC* |
| *Ugdh-F* | *CAACAGCGATTGGAATGGACC* |
| *Ugdh-R* | *TCTGGCAAATTCAGAGCCTCA* |
